# Expansion of circulating stem-like CD8^+^ T cells by adding CD122-directed IL-2 complexes to radiation and anti-PD1 therapies in mice

**DOI:** 10.1038/s41467-023-37825-x

**Published:** 2023-04-12

**Authors:** Kateryna Onyshchenko, Ren Luo, Elena Guffart, Simone Gaedicke, Anca-Ligia Grosu, Elke Firat, Gabriele Niedermann

**Affiliations:** 1 Department of Radiation Oncology, Faculty of Medicine, University of Freiburg, Freiburg, Germany; 2 Faculty of Biology, University of Freiburg, Freiburg, Germany; 3 Laboratory of Biosynthesis of Nucleic Acids, Institute of Molecular Biology and Genetics of NASU, Kyiv, Ukraine; 4 German Cancer Consortium (DKTK), Partner Site Freiburg, Freiburg, Germany; 5 German Cancer Research Center (DKFZ), Heidelberg, Germany; 6 Division of Thoracic Tumor Multimodality Treatment, Cancer Center, West China Hospital, Sichuan University, Chengdu, Sichuan China

**Keywords:** Radiotherapy, Interleukins, Cytotoxic T cells, Cancer immunotherapy, Tumour immunology

## Abstract

Combination of radiation therapy (RT) with immune checkpoint blockade can enhance systemic anti-tumor T cell responses. Here, using two mouse tumor models, we demonstrate that adding long-acting CD122-directed IL-2 complexes (IL-2c) to RT/anti-PD1 further increases tumor-specific CD8^+^ T cell numbers. The highest increase (>50-fold) is found in the blood circulation. Compartmental analysis of exhausted T cell subsets shows that primarily undifferentiated, stem-like, tumor-specific CD8^+^ T cells expand in the blood; these cells express the chemokine receptor CXCR3, which is required for migration into tumors. In tumor tissue, effector-like but not terminally differentiated exhausted CD8^+^ T cells increase. Consistent with the surge in tumor-specific CD8^+^ T cells in blood that are migration and proliferation competent, we observe a CD8-dependent and CXCR3-dependent enhancement of the abscopal effect against distant/non-irradiated tumors and find that CD8^+^ T cells isolated from blood after RT/anti-PD1/IL-2c triple treatment can be a rich source of tumor-specific T cells for adoptive transfers.

## Abstract (web summary)

IL-2 complexes (IL-2c) which are IL-2 complexed with anti-IL-2 can be used to promote T cell function. Here authors use IL2c in addition to checkpoint inhibitors and irradiation to increase anti-tumour T cell responses and promote tumour rejection in mouse cancer models.

## Introduction

Chronic exposure to antigen induces a dysfunctional, exhausted state of tumor-infiltrating lymphocytes (TILs), which is characterized by upregulation of multiple co-inhibitory receptors, including PD1, and progressive loss of effector functions and proliferation^1,2^. This differentiation into exhausted cells starts from relatively undifferentiated TCF1^+^PD1^+^ progenitor cells which are stem cell-like with the ability to self-renew and differentiate into TCF1^-^TIM3^+^PD1^+^ cells^3,4^. Without treatment or upon PD1/PD-L1 immune checkpoint blockade (ICB), differentiation proceeds through a transitory/intermediate stage with emerging effector functions to the PD1^high^ terminally differentiated state^5,6^. Exhausted T cell subsets have also been characterized following tumor irradiation^7,8^. PD1/PD-L1 blockade can reinvigorate the exhausted T cell response apparently largely through expansion of TCF1^+^ stem-like progenitors^3,4,6,9^. Since only some patients respond to PD1/PD-L1 ICB, it is however necessary to find more effective combinations, for example, with conventional cytotoxic therapies or other immunotherapeutics.

Radiation therapy (RT) is a localized cytotoxic therapy; hypofractionated RT, as used here, can induce tumor-specific CD8^+^ T cells by promoting dendritic cell cross-priming. These T cells contribute not only to local control of irradiated tumors, but circulating T cells can also trigger regression of non-irradiated lesions (the so-called “abscopal effect”)^10,11^. However, strong abscopal responses are observed only in some patients even when RT is combined with ICB^10–14^. Thus, further enhancement of RT-induced systemic antitumor T cell responses would be highly desirable.

Also of great interest are dual immunotherapy combinations of anti-PD1/PD-L1 and interleukin-2 (IL-2) or IL-2 derivatives. The pro-proliferative effect of IL-2 on T cells was discovered decades ago^15^. However, clinical use of recombinant IL-2 (rIL-2) is limited because frequent, high doses are usually required, causing vascular toxicity that is potentially fatal. Moreover, rIL-2 expands immunosuppressive CD4^+^ regulatory T cells (Tregs), which are detrimental to a successful anti-tumor T cell response. Vascular toxicity and expansion of immunosuppressive CD4^+^ Tregs depend on the binding of rIL-2 to the trimeric alpha (CD25)/beta (CD122)/gamma (CD132) IL-2 receptor, the high-affinity IL-2 receptor that is expressed not only transiently by recently activated effector T cells but also constitutively by CD4^+^ Tregs and vascular endothelial cells^16^. Great efforts are being made to develop derivatives that are longer acting, less toxic, and less immunosuppressive than rIL-2^17–22^. These include derivatives targeting the intermediate-affinity CD122/CD132 IL-2 receptor on activated CD8^+^ T cells and NK cells. The CD122-directed IL-2/anti-IL-2 complexes (IL-2c) used in the present study prototypically represent long-acting CD122-directed IL-2 derivatives^23^.

Dual treatment with anti-PD-L1 and low dose rIL-2 can synergistically increase virus-specific CD8^+^ T cells and reduce virus titers in chronically infected mice^24^. In line with this, preclinical and early clinical trials indicated promising antitumor effects for dual combinations of ICB and IL-2 derivatives^19,25–28^. Moreover, recent mouse studies suggest that triple combinations with additional RT could further enhance the antitumor effects. In models with a single irradiated tumor, better responses to triple treatment containing tumor vasculature-targeted L19-IL-2^29^ or CD122-preferential pegylated IL-2^30^ were reported; the latter study also reported better control of lung micrometastases. However, it is not yet known whether such triple treatment can improve the RT-induced abscopal effect against established non-irradiated tumor lesions as compared to double combinations.

Currently, little is known about how effectively triple combinations containing an IL-2 derivative expand tumor-specific T cells, and what effects they have on subsets of dysfunctional/exhausted T cells in different compartments relevant to the antitumor T cell response. Here, using two mouse tumor models, we show that adding CD122-directed IL-2c to RT and anti-PD1 can greatly expand tumor-specific CD8^+^ T cells. The greatest expansion was observed in extra-tumoral compartments, particularly in blood. There was primarily expansion of stem-like cells and, in tumor tissue, effector-like transitory, but not terminally exhausted cells. Very pronounced and also greatest in blood was the increase in stem-like cells expressing CXCR3, a chemokine receptor important for T cell migration into tumors^31^. In line with the strong expansion of stem-like CXCR3^+^ tumor-specific CD8^+^ T cells in the blood circulation, CXCR3 blockade abrogated the abscopal effect in mice treated with RT/anti-PD1/IL-2c. Moreover, this triple combination more potently than RT/anti-PD1 or RT/IL-2c induced abscopal regression in two abscopal mouse models with established contralateral flank tumors. In addition, blood from triple-treated mice proved to be an effective source of T cells for adoptive T cell transfer (ACT).

## Results

### Triple treatment preferentially triggers proliferation of antitumor CD8^+^ T cells in extratumoral compartments

**Fig. 1:**
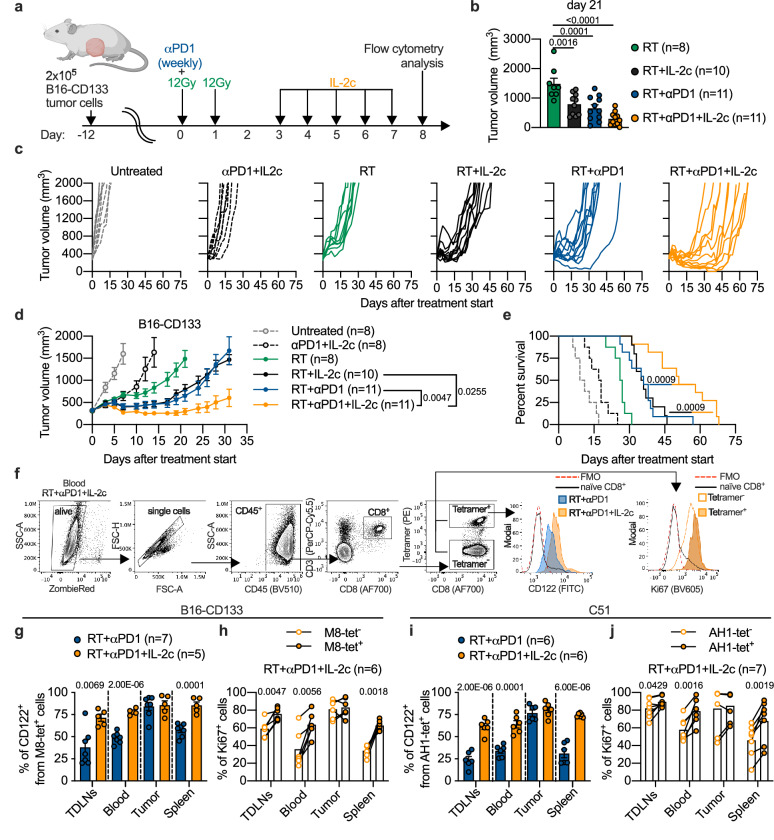
Adding CD122-directed IL-2c to RT + αPD1 improves antitumor efficacy correlating with enhanced proliferation of tumor-specific CD8^+^ T cells. **a** Scheme for treatments and T cell analyses. **b** Comparison of tumor volume between RT and combination treatments at d21 after treatment start (*n* = 8‒11 mice per group). **c** Individual tumor growth curves for irradiated B16-CD133 tumors. Tumor growth (**d**), and survival (**e**) of B16-CD133 tumor-bearing mice (*n* = 8‒11 mice per group). **f**, Gating strategy to determine CD122^+^ and Ki67^+^ tumor-specific T cells. Percentage of CD122^+^ among tetramer-positive CD8^+^ T cells in RT + αPD1 (blue) and RT + αPD1 + IL-2c (orange) treatment groups in indicated compartments of B16-CD133 (**g**) and C51 (**i**) tumor-bearing mice (*n* = 5–7 mice per group). Percentage of Ki67^+^ among tetramer-positive and tetramer-negative cells after RT + αPD1 + IL-2c treatment in B16-CD133 (**h**) and C51 (**j**) tumor model (B16-CD133, *n* = 6; C51, *n* = 7). Data were collected from 2 to 3 independent experiments for each treatment group. Symbols represent individual mice (**b**, **g**–**j**). *P* values are shown and error bars indicate mean ± SEM; statistical comparisons were performed using one-way ANOVA with Dunnet’s multiple comparisons test (**b**, **d**), log-rank test (**e**), two-tailed unpaired Student’s *t* test (**g**, **i**) or two-tailed paired Student’s *t* test (**h**, **j**). Source data are provided as a Source Data file.

We initially performed experiments in mouse models with only one tumor (Fig. 1a and Supplementary Fig. 1a, b). As shown in Fig. 1b–e, dual anti-PD1/IL-2c immunotherapy and RT monotherapy were only slightly effective in mice bearing a B16-CD133 melanoma tumor. Of the three RT/IT combinations, the RT/anti-PD1/IL-2c triple combination was most effective in controlling tumor growth and extending survival (Fig. 1b–e). In the C51 colon carcinoma model, which in contrast to B16-CD133 is a cold tumor model (Supplementary Fig. 2a–d), triple treatment also improved tumor control and survival compared with RT/anti-PD1 (Supplementary Fig. 1c–e). However, compared with RT/IL-2c, survival differences did not reach statistical significance (Supplementary Fig. 1d). In the C51 model, a considerable number of mice either triple-treated or treated with RT + IL-2c showed complete tumor regression and were re-challenged with tumor cells 120 days thereafter. All mice, except two triple-treated ones, which showed a delayed relapse, resisted the re-challenge (Supplementary Fig. 1f, g).

We determined CD122 expression and proliferation of tumor-specific CD8^+^ T cells using the M8 and the AH1 MHC-I tetramer for the B16-CD133 and the C51 model, respectively (Fig. 1f–j, and Supplementary Fig. 3a, b). These analyses, as most others, were done on d8 after treatment start. As shown in Fig. 1f, g, i, triple treatment induced more CD122^+^ tumor-specific CD8^+^ T cells than RT/anti-PD1 in tumor-draining lymph nodes (TDLNs), blood, and spleen in both the B16-CD133 and the C51 models. Moreover, in these extratumoral compartments, tetramer-positive (tet^+^) tumor-specific CD8^+^ T cells more strongly proliferated than tetramer-negative (bulk) T cells (Fig. 1f, h, j). These data thus demonstrated a proliferation advantage for the tumor-specific over bulk CD8^+^ T cells in secondary lymphatic organs and blood of triple-treated mice associated with an increase in CD122-expressing cells. Moreover, they demonstrated that systemic immune responses are crucial for the benefit of triple treatment consisting of local RT, anti-PD1, and CD122-directed IL-2c.

### Largest increase in triple treatment-induced antitumor CD8^+^ T cells in blood

**Fig. 2:**
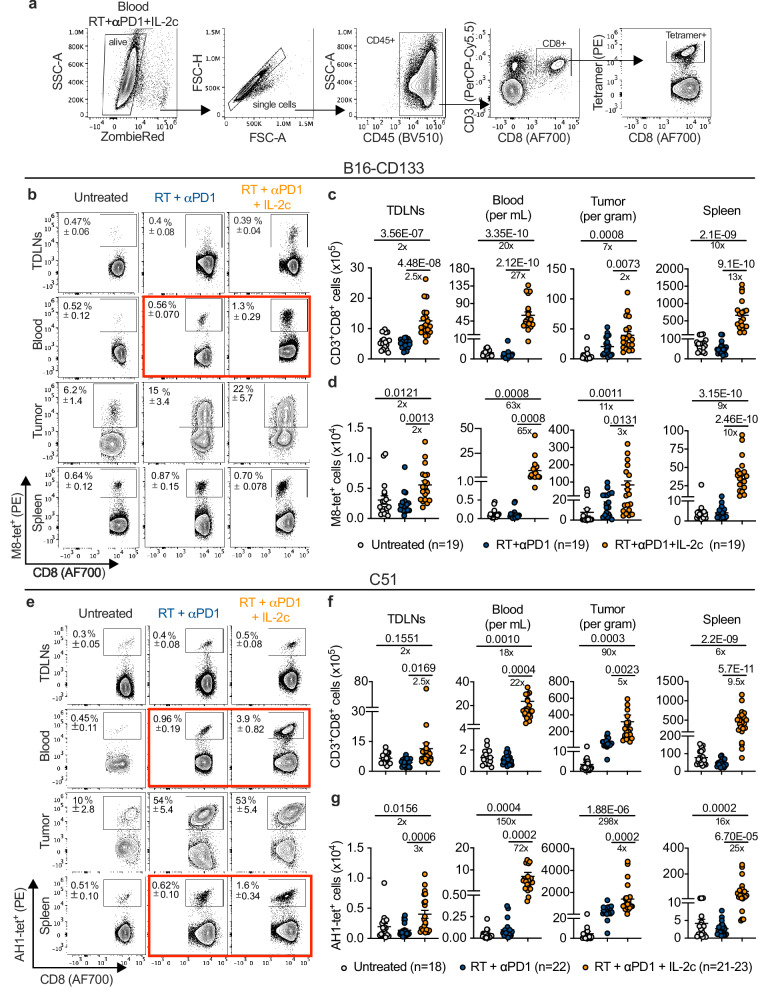
RT + αPD1 + IL-2c dramatically expands tumor-specific CD8^+^ T cells in the B16-CD133 melanoma and the C51 colon carcinoma model. **a** Gating strategy to identify tumor-specific tetramer^+^ CD8^+^ T cells. Representative flow cytometry plots that also show the mean ± SEM of the frequency of tetramer-positive CD8^+^ T cells in TDLNs, blood, tumor, and spleen for indicated treatment groups in the B16-CD133 (**b**), and the C51 (**e**) tumor model at d8 after treatment start. **c**, **f**, Absolute numbers of CD8^+^ T cells, **d**, **g**, absolute numbers of tetramer-positive CD8^+^ T cells in TDLNs, blood, tumor, and spleen in the B16-CD133 and the C51 tumor model. Data were collected from at least six independent experiments with 2‒4 mice in each treatment group (*n* = 18‒23 individual mice). *P* values are shown and error bars indicate mean ± SEM; statistical comparisons were performed using one-way ANOVA with Tukey’s multiple comparisons test. Source data are provided as a Source Data file.

Adding IL-2c increased the absolute numbers of tumor-specific CD8^+^ T cells in all compartments analyzed (Fig. 2a–g). The smallest increase was observed in TDLNs (2- and 3-fold in triple-treated vs. RT/anti-PD1-treated mice in the B16-CD133 and the C51 models, respectively). In the irradiated tumors, tumor-specific CD8^+^ T cells increased threefold (B16-CD133 model) and fourfold (C51 model). In the spleen, the increase was 10- and 25-fold, respectively. Of note, the largest difference was observed in the blood where tumor-specific CD8^+^ T cells increased in triple-treated vs. RT/anti-PD1-treated mice 65-fold in the B16-CD133 model and 72-fold in the C51 model; in line with the Ki67 data in Fig. 1h, j, tumor-specific CD8^+^ T cells (Fig. 2d, g) increased more than total CD8^+^ T cells (Fig. 2c, f). In both tumor models, the surge in absolute numbers of tumor-specific CD8^+^ T cells in blood was associated with a significant increase in the proportion of tet^+^ CD8^+^ T cells (Fig. 2b, e, boxed in red; Supplementary Fig. 3d, f).

### Antitumor effects of triple treatment depend on TDLNs

**Fig. 3:**
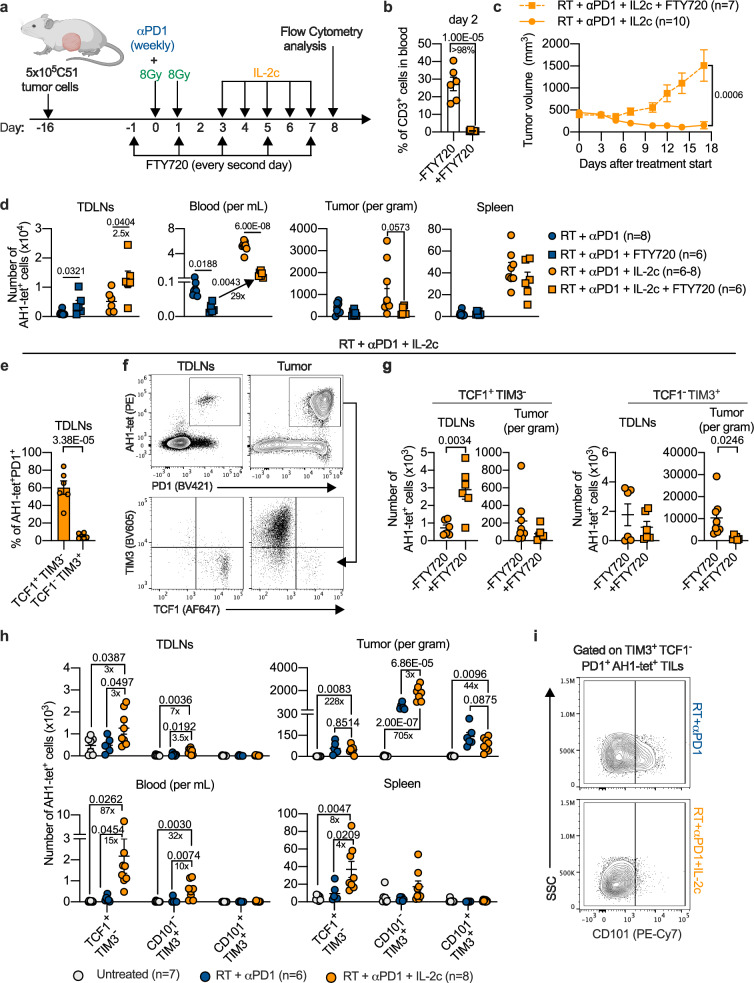
RT + αPD1 + IL-2c treatment expands stem-like exhausted T cells in the periphery and increases effector-like transitory cells in tumor tissue. **a** Scheme of treatment and T cell analysis. **b** Percentage of CD3^+^ T cells in the blood of mice with (*n* = 6) or without (*n* = 7) FTY720 treatment. **c** Mean tumor growth curves for triple-treated mice with (*n* = 7) or without (*n* = 10) FTY720. **d** Number of AH1-tetramer^+^ (AH1-tet^+^) cells in RT + αPD1 and RT + αPD1 + IL-2c treatment groups with (*n* = 6) and without (*n* = 6‒8) FTY720. **e** Percentage of TCF1^+^TIM3^-^ and TCF1^-^TIM3^+^ cells among tumor-specific PD1^+^CD8^+^ T cells in the TDLNs of triple-treated mice (*n* = 6). **f** Representative flow cytometry plots for the characterization of TCF1^+^TIM3^-^ and TCF1^-^TIM3^+^ cells among tumor-specific PD1^+^CD8^+^ T cells in the TDLNs vs. tumor. **g** Number of TCF1^+^TIM3^-^ (left) and TCF1^-^TIM3^+^ (right) tumor-specific PD1^+^CD8^+^ T cells in TDLNs and tumors of mice that received triple treatment with or without FTY720 (*n* = 6‒8). **h** Numbers of tumor-specific TCF1^+^TIM3^-^PD1^+^ (stem-like), CD101^-^TIM3^+^ TCF1^-^PD1^+^ (effector-like transitory), and CD101^+^TIM3^+^TCF1^-^PD1^+^ (terminally exhausted) cells (*n* = 6‒8). **i** Representative flow cytometry plots for CD101^+^ and CD101^-^ cells among tumor-specific TIM3^+^TCF1^-^PD1^+^ CD8^+^ TILs. Data were collected from at least two independent experiments for each treatment group. Symbols represent individual mice (**b**, **d**, **e**, **g**, **h**). *P* values are shown and error bars indicate mean ± SEM; statistical comparisons were performed using two-tailed unpaired Student’s *t* test (**b**–**g**), or one-way ANOVA with Tukey’s multiple comparisons test (**h**). Source data are provided as a Source Data file.

We used FTY720 to block the sphingosine 1-phosphate receptor, thereby trapping T cells in lymphoid organs^32^ (Fig. 3a). The FTY720 blockade was confirmed by flow cytometry one day before the start of IL-2c injections (Fig. 3b). FTY720 blockade strongly abrogated the antitumor effect of RT/anti-PD1/IL-2c triple treatment, indicating that tumor-resident T cells were not sufficient for tumor control (Fig. 3c). In addition, tumor-specific CD8^+^ T cells accumulated in TDLNs (Fig. 3d), consistent with the view that RT induces tumor-specific CD8^+^ T cells in lymph nodes draining irradiated tumors^33,34^; in blood, tumor-specific CD8^+^ T cells decreased but, under FTY720 blockade, they still expanded 29-fold in triple-treated vs. RT/anti-PD1-treated mice (Fig. 3d). This may indicate that even though TDLNs were the primary source of tumor-specific T cells required for successful tumor control, the expansion of these cells took place mostly in blood.

Recent findings have demonstrated that stem-like TCF1^+^PD1^+^ CD8^+^ T cells are crucial for the response to anti-PD1/PD-L1^3,4,6,9^ and that TDLNs are important for the RT-induced abscopal effect^33,34^. Therefore, we next focused on this most immature subset of exhausted T cells. In TDLNs of triple-treated mice more than 50% of AH1-tet^+^ CD8^+^ T cells had a stem-like phenotype (TCF1^+^TIM3^-^PD1^+^) (Fig. 3e, f). Almost all tetramer^+^ TILs were PD1^+^, which is consistent with previous findings^35,36^. FTY720 administration caused accumulation of stem-like tumor-specific CD8^+^ T cells in TDLNs and reduced the numbers of more differentiated TCF1^-^TIM3^+^PD1^+^ cells in the tumor (Fig. 3g). FTY720 did not change the percentage of these subpopulations (Supplementary Fig. 4), indicating that the changes in absolute numbers were largely due to blockade of T cell egress from the lymph nodes. Taken together, these data suggested that triple treatment required TCF1^+^ cells from TDLNs which acted as a source for more differentiated CD8^+^ T cells in the tumor.

### Adding IL-2c to RT/anti-PD1 most strongly expanded the stem-like subset of exhausted CD8^+^ T cells

Triple treatment preferentially expanded cells with markers of stem-like exhausted T cells (TCF1^+^TIM3^-^PD1^+^) and, to a lesser degree, cells with markers of transitory exhausted T cells (CD101^-^TCF1^-^TIM3^+^PD1^+^) but not cells with markers of terminally exhausted cells (CD101^+^TCF1^-^TIM3^+^PD1^+^) (Fig. 3h). Stem-like tumor-specific CD8^+^ T cells increased the most in peripheral compartments. In the C51 model, they had expanded in blood 15-fold and 87-fold compared to RT/anti-PD1 and untreated animals, respectively, on d8 after treatment start. In contrast, in the irradiated tumor, effector-like CD101^-^TCF1^-^TIM3^+^PD1^+^ cells, which in chronic viral infection have been shown to constitute a transitory population between stem-like and terminally exhausted cells^3^, increased most (threefold) compared to RT/anti-PD1, whereas stem-like cells did not further increase compared to RT/anti-PD1 (Fig. 3h, i). Interestingly, we still found stem-like tumor-specific CD8^+^ T cells two and three weeks after treatment start in all compartments analyzed, although the absolute numbers were lower in TDLNs at the 3-week time point (Supplementary Fig. 5a–d).

We performed an in vitro experiment with sorted CD101^-^TIM3^-^PD1^+^CD8^+^ T cells from the peripheral blood and CD101^-^TIM3^+^PD1^+^CD8^+^ TILs of triple-treated mice. The sorted cells were co-cultured with C51 tumor cells that had been pre-treated with IFNγ to upregulate PD-L1; the CD8^+^ T cell phenotype was flow-cytometrically analyzed 3 days later. As shown in Supplementary Fig. 6, the majority of the AH1-tet^+^ blood-derived CD101^-^TIM3^-^ cells had converted to TIM3^+^ cells, a proportion of which also upregulated CD101. Only a minority of blood-derived AH1-tet^-^ cells upregulated TIM3 and CD101. Both AH1-tet^+^ and AH1-tet^-^ TILs had increased TIM3 expression and some of the cells up-regulated CD101. The CD101 upregulation on the AH1-tet^-^ TILs is likely due to the presence of other than AH1-specific tumor-reactive CD8^+^ T cells among the sorted PD1^+^CD8^+^ TILs (Supplementary Fig. 6). These data showed that CD101^+^ tumor-specific CD8^+^ T cells can arise from CD101^-^ cells.

CD28 is the main positive costimulator of T cell activation and is required for CD8 T cell proliferation after PD1 blockade in mice chronically infected with LCMV^37^. Consistent with the most pronounced expansion of (stem-like) tumor-specific CD8^+^ T cells in blood (Fig. 2d, g and Fig. 3h), we found the strongest expansion (>10-fold) of CD28^+^ tumor-specific CD8^+^ T cells also in blood of triple-treated vs. RT/anti-PD1-treated mice (Supplementary Fig. 7a–c).

### IL-2c increased effector functions of tumor-specific TILs

**Fig. 4:**
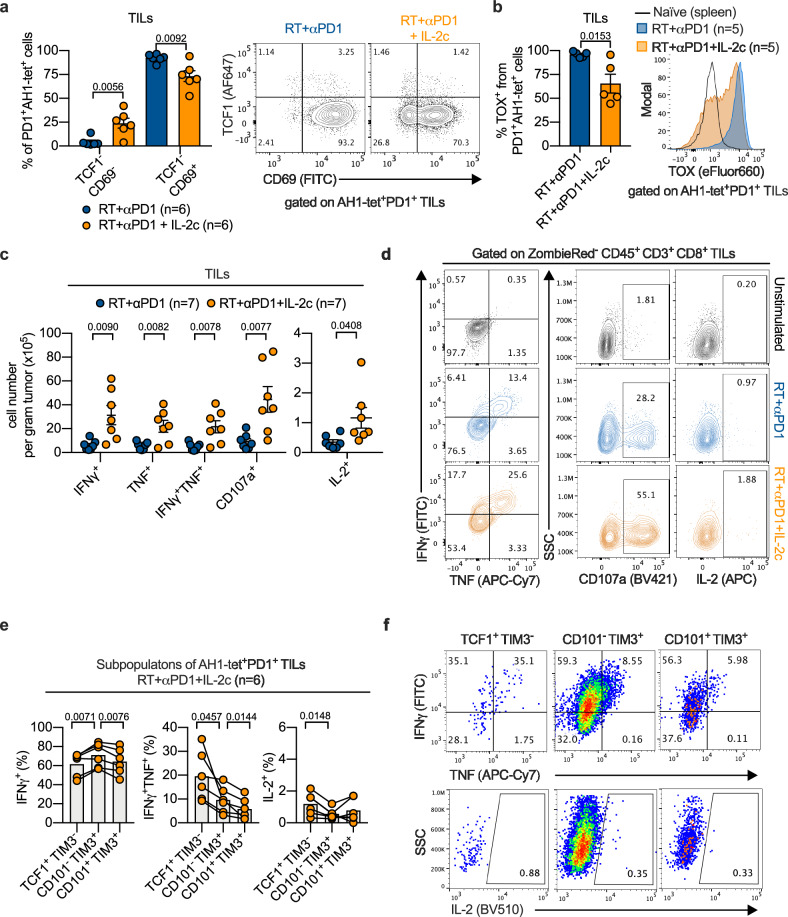
IL-2c improve the effector functions of tumor-specific T cells. **a** Percentage (left), and representative flow cytometry plots (right) of TCF1^-^CD69^-^PD1^+^ and TCF1^-^CD69^+^PD1^+^ tumor-specific CD8^+^ TILs (*n* = 6). **b** Percentage (left) and representative flow cytometry plot (right) of TOX^+^ in tumor-specific PD1^+^CD8^+^ TILs (*n* = 5). Numbers (**c**), and representative flow cytometry plots (**d**) of IFNγ^+^, TNF^+^, IFNγ^+^TNF^+^, CD107a^+^, and IL-2^+^ CD8^+^ T cells after ex vivo stimulation with AH1 peptide (*n* = 7). Percentage (**e**) and representative flow cytometry plots (**f**) of cytokine-producing cells among different subpopulations of AH1-tet^+^PD1^+^ TILs of RT + αPD1 + IL-2c-treated mice (*n* = 6). Data were collected from three independent experiments for each treatment group. Symbols represent individual mice. *P* values are shown and error bars indicate mean ± SEM; statistical comparisons were performed using two-tailed unpaired Student’s *t* test (**a**–**c**), or two-tailed paired Student’s *t* test (**e**). Source data are provided as a Source Data file.

We confirmed the increase in effector-like CD8^+^ TILs in triple-treated vs. RT/anti-PD1-treated mice using markers based on an alternative nomenclature of T cell exhaustion considering the residency marker CD69 ^6^. Whereas the proportion of effector-like (TCF1^-^CD69^-^PD1^+^) intermediate exhausted TILs increased, the proportion of terminally exhausted (TCF1^-^CD69^+^ PD1^+^) tumor-specific CD8^+^ TILs was reduced (Fig. 4a). The critical exhaustion regulator Tox^38–40^, and T-bet, known to upregulate effector functions^41^, are key antagonistic regulators of exhaustion. Whereas T-bet is crucial for transitioning into the intermediate exhausted state, Tox expression is reduced during this state^6^. Consistent with that, we found in triple-treated mice fewer Tox-expressing (Fig. 4b, Supplementary Fig. 8a) and more T-bet-expressing tumor-specific CD8^+^ T cells (Supplementary Fig. 8b, c), and more TILs secreting the effector cytokines IFNγ and TNF including more polyfunctional IFNγ^+^TNF^+^ TILs, as well as cells positive for CD107a, a degranulation marker of cytotoxic T cells (Fig. 4c, d, and Supplementary Fig. 8d). In the C51 model, the percentage of IFNγ^+^TNF^+^ TILs at d8 after treatment start was 23 ± 5 (mean ± SEM) % in triple-treated mice whereas it was 10 ± 2 (mean ± SEM) % in RT/anti-PD1-treated mice (Supplementary Fig. 8d). Analysis of tetramer^+^ TILs from triple-treated mice in both tumor models showed that the percentage of polyfunctional (IFNγ^+^TNF^+^) cells decreased from the TCF1^+^TIM3^-^PD1^+^ to the CD101^-^TCF1^-^TIM3^+^PD1^+^ to the CD101^+^TCF1^-^TIM3^+^PD1^+^ subset (Fig. 4e, f; Supplementary Fig. 8e, f). In all these three TIL subsets, IL-2-producing cells were barely detectable (Fig. 4e, f; Supplementary Fig. 8e, f). In contrast, the percentages of IFNγ^+^TNF^+^ and of IL-2^+^ cells were much higher in an in vitro-induced peptide-specific T cell line (Supplementary Fig. 8e, f). Decreasing IFNγ/TNF co-production and virtual absence of IL-2 production by TIL subsets with markers of stem-like, transitory, and terminally exhausted cells in triple-treated mice are consistent with some degree of dysfunction/exhaustion^42^.

### IL-2c strongly increased CXCR3^+^ stem-like tumor-specific CD8^+^ T cells

**Fig. 5:**
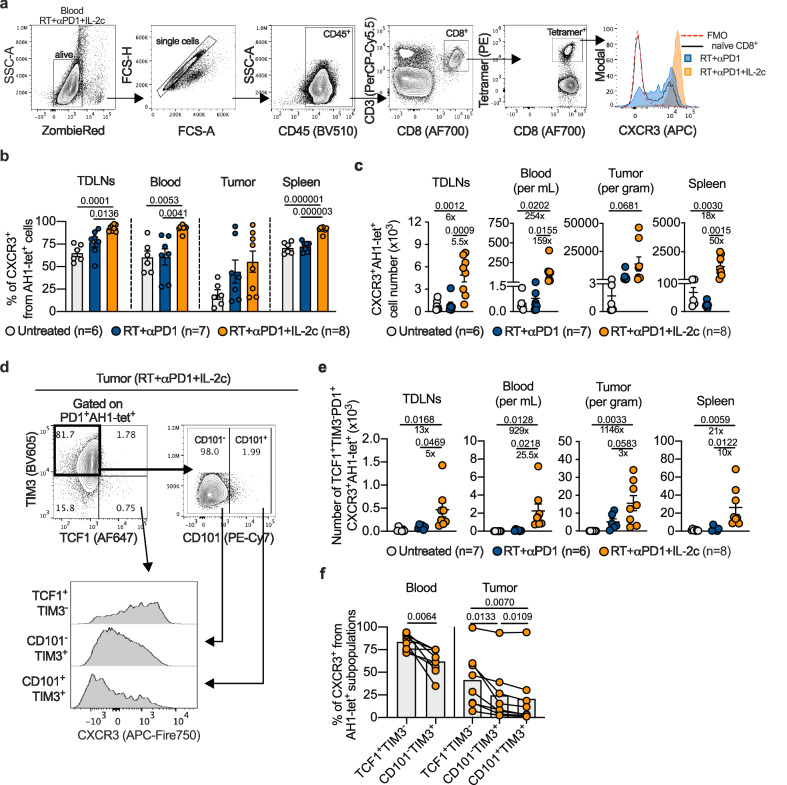
IL-2c increase CXCR3 expression and expand CXCR3+ TCF1+TIM3- tumor-specific CD8^+^ T cells. **a** Gating strategy to determine CXCR3^+^ tumor-specific T cells. Percentage (**b**) and numbers (**c**) of CXCR3^+^ AH1-tet^+^ cells in TDLNs, blood, tumor, and spleen of mice treated as indicated (*n* = 6‒8). **d** Gating strategy to determine CXCR3^+^ expression of different subpopulations of AH1-tet^+^PD1^+^ CD8^+^ T cells. **e** Number of CXCR3^+^AH1-tet^+^ stem-like (TCF1^+^TIM3^-^PD1^+^) cells in TDLNs, blood, tumor and spleen of C51 tumor-bearing mice (*n* = 6‒8). **f** Progressive decrease in CXCR3 expression in blood- or tumor-derived AH1-tet^+^ CD8^+^ T cell subsets with markers of the exhaustion lineage in triple-treated mice (*n* = 8). Data were collected from three independent experiments for each treatment group. Symbols represent individual mice. *P* values are shown and error bars indicate mean ± SEM; statistical comparisons were performed using one-way ANOVA with Tukey’s multiple comparisons test (**b**, **c**, **e**), or two-tailed paired Student’s *t* test (**f**). Source data are provided as a Source Data file.

The CXCR3 chemokine receptor and its ligands CXCL9/10/11 are crucial for the attraction of tumor antigen-specific T cells into tumor tissue^31^. Following triple treatment, the percentage and absolute number of CXCR3^+^ tumor-specific CD8^+^ T cells increased strongly in extratumoral compartments, particularly in blood (Fig. 5a–c, and Supplementary Fig. 9a, b). Compared to RT/anti-PD1 treatment, blood samples from triple-treated mice showed a more than 150-fold increase in CXCR3^+^ tumor-specific CD8^+^ T cells (Fig. 5c and Supplementary Fig. 9b).

Subpopulation analyses of tumor-specific CD8^+^ T cells showed that not only total, but also stem-like CXCR3^+^ cells expanded (Fig. 5d, e). CXCR3 expression markedly dropped with increasing differentiation, as we show for subsets with exhaustion lineage markers in blood as well as tumor tissue (Fig. 5d, f). This drop is in line with *CXCR3* expression changes in subsets of exhausted cells in mice chronically infected with LCMV^5^ or bearing B16-OVA tumors^4^ as indicated by our analysis of publicly available data sets (Supplementary Fig. 9c).

Analyses of The Cancer Genome Atlas (TCGA) data sets showed that high tumor expression of *CXCR3* correlated with better survival of melanoma patients with a hazard ratio of 0.59 (Supplementary Fig. 9d). Moreover, *CXCR3* expression correlated with expression of the IL-2 receptor beta *IL2RB* (CD122) gene (Supplementary Fig. 9e). Together with our experimental data, this suggests that treatment with CD122-directed IL-2 derivatives can promote the expression of CXCR3 which has an important function in antitumor responses in mice and humans.

### Triple treatment enhanced the abscopal effect against established non-irradiated tumors depending on CD8^+^ and CXCR3^+^ cells

**Fig. 6:**
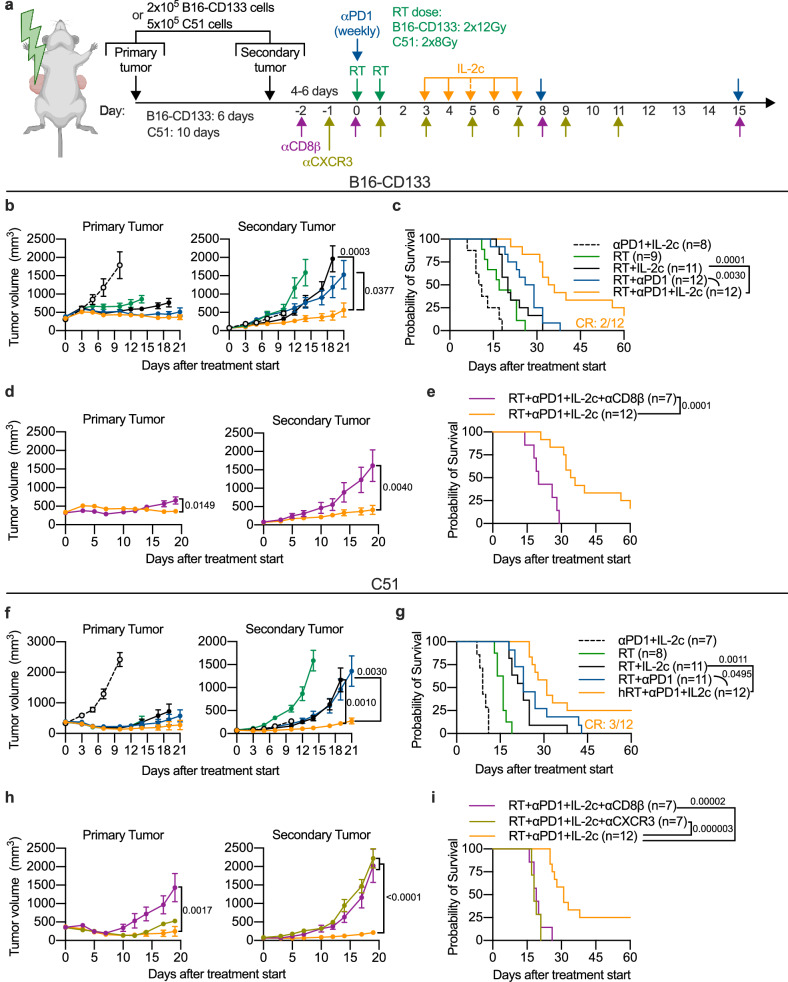
Local and abscopal effects of RT combined with IL-2c and αPD1 depend on CD8^+^ and CXCR3+ cells. **a** Scheme for treatments. B16-CD133 (*n* = 8‒12) (**b**), and C51 (*n* = 7‒12) (**f**) tumor growth of irradiated (left) and non-irradiated (right) tumors. Survival of mice bearing B16-CD133 (**c**), or C51 (**g**) tumors. **d**, **e**, CD8^+^ cell depletion (*n* = 7) in the B16-CD133 tumor model (CR, complete response; indicates how many mice were cured). **h**, **i**, CD8^+^ cell depletion (*n* = 7) and CXCR3 blockade (*n* = 7) in the C51 tumor model. Data were collected from 2 to 3 independent experiments for each treatment group. *P* values are shown and error bars indicate mean ± SEM; statistical comparisons were performed using two-tailed Student’s *t* test (**b**, **d**, **f**, **h**), or log-rank test (**c**, **e**, **g**, **i**). Source data are provided as a Source Data file.

Since the triple treatment caused a strong expansion of CXCR3^+^ tumor-specific CD8^+^ T cells in the blood circulation, we examined the effect of triple treatment on the abscopal effect in mice with two contralateral tumors, where only the primary was irradiated (Fig. 6a).

In both tumor models, triple treatment resulted in significantly better abscopal tumor control and survival than all double treatments (RT/anti-PD1, RT/IL-2c, and anti-PD1/IL-2c) (Fig. 6b, c, f, g and Supplementary Fig. 10a–f). Moreover, complete cures were found only in triple-treated mice (Fig. 6c, g), and all of these mice were protected against re-challenge with tumor cells (Supplementary Fig. 11a–c). No statistically significant difference was observed between RT/anti-PD1 and RT/IL-2c (Fig. 6b, c, f, g), both of which induced better abscopal tumor control than RT alone (Supplementary Fig. 10b, e).

By concomitantly adding either CD8β-depleting or CXCR3-blocking antibodies to triple treatment, we found that both CD8^+^ and CXCR3^+^ cells were crucial for the abscopal effect as adding these antibodies completely attenuated abscopal tumor control and significantly worsened mouse survival (Fig. 6d, e, h, i). To identify CXCL9/10-secreting cell populations in tumors of triple-treated mice, we performed a flow cytometry experiment where myeloid cells turned out to be important CXCL9/10 producers, including in the unirradiated tumor (Supplementary Fig. 12a–d), consistent with previous findings^43–46^.

### Blood of triple-treated mice contained polyfunctional non-apoptotic antitumor CD8^+^ T cells

Tumor-specific CD8^+^ T cells with high proliferative capacity in blood, including the strongly expanded TCF1^+^PD1^+^ stem-like and perhaps also the emerging TCF1^-^CD101^-^TIM3^+^ population (see Fig. 3h), may not only be important for therapeutic effects against distant abscopal tumors, but may also be very useful for ACT.

**Fig. 7:**
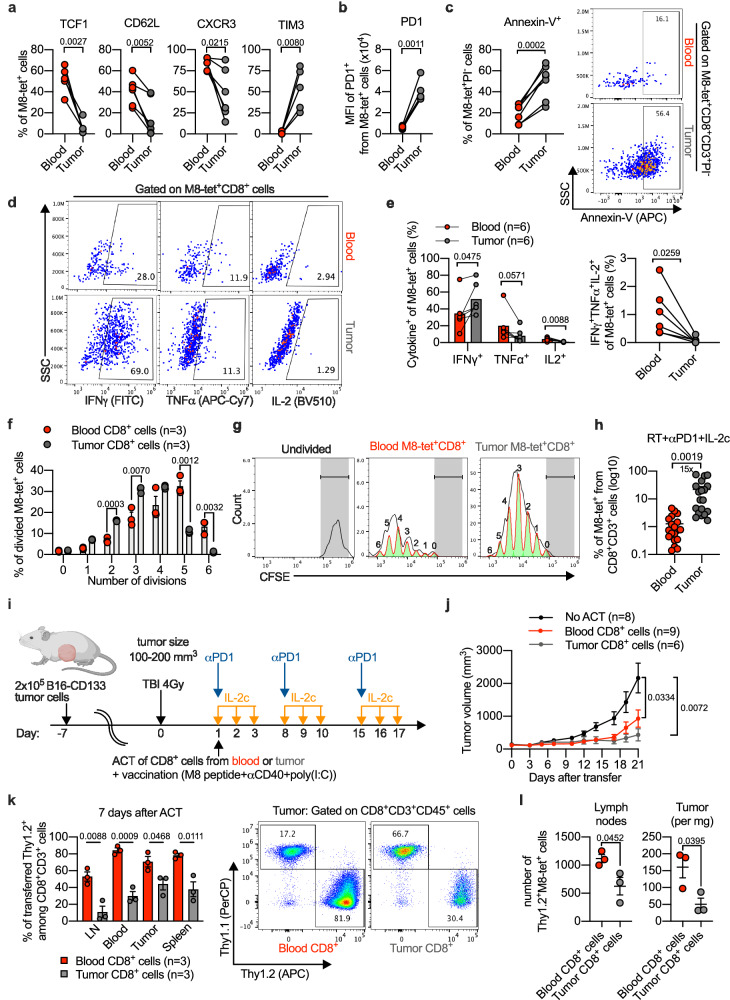
Adoptive transfer of CD8^+^ T cells isolated from blood or tumors of RT + αPD1 + IL-2c-treated mice in the B16-CD133 model. **a**, **b** Flow cytometry analysis of M8-tet^+^ CD8^+^ T cells from blood and tumor after RT + αPD1 + IL-2c treatment (*n* = 5‒6). **c** Flow cytometry analysis and representative example of the early apoptotic (Annexin-V^+^) population among M8-tet^+^PI^-^ CD8^+^ T cells in blood and tumor (*n* = 6). Representative flow cytometry plots (**d**) and percentage (**e**) of IFNγ^+^, TNF^+^, IL-2^+^ (left), and IFNγ^+^TNF^+^IL-2^+^ polyfunctional cells (right) among M8-tet^+^CD8^+^ T cells from blood and tumor after 4 h ex vivo stimulation with PMA plus ionomycin (*n* = 6). Percentage (**f**) and representative CFSE intensity histograms (**g**) of dividing M8-tet^+^CD8^+^ T cells from blood or tumors of triple-treated mice stimulated ex vivo with αCD3 + αCD28 (*n* = 3). **h** Percentages of M8-tet^+^ among CD8^+^ T cells in blood vs. tumor of mice treated with RT + αPD1 + IL-2c at d8 after treatment start (*n* = 19). **i** Scheme of adoptive T cell transfer (ACT) experiment. **j** B16-CD133 tumor growth in recipient mice after ACT of CD8^+^ T cells isolated from blood (*n* = 9) or tumors (*n* = 6) of donor mice, or without cell transfer (*n* = 8). **k** Percentage (left) and representative flow cytometry plots (right) of transferred blood- or tumor-derived Thy1.2^+^ CD8^+^ T cells among total CD8^+^ T cells in recipient mice at d7 after ACT (*n* = 3). **l** Numbers of recovered donor tumor-specific CD8^+^ T cells (blood- vs. tumor-derived) in LN and tumor of recipient mice at d7 after ACT (*n* = 3). Data are from 2 to 3 independent experiments (**a**–**e**, **j**), or one of two independent experiments (**f**, **k**, **l**). Symbols represent individual mice (**a**–**c**, **e**, **h**, **k**, **l**), or wells (**f**). *P* values are shown and error bars indicate mean ± SEM; statistical comparisons were performed using two-tailed paired Student’s *t* test (**a**–**e**, **h**), two-tailed unpaired Student’s *t* test (**f**, **k**–**l**), or one-way ANOVA with Tukey’s multiple comparisons test (**j**). Source data are provided as a Source Data file.

As presented in Fig. 7a (for the B16-CD133 tumor model) and Supplementary Fig. 13a (for the C51 tumor model), a direct comparison of tumor-specific CD8^+^ T cells from peripheral blood and irradiated tumor of triple-treated mice showed that blood contained more tumor-specific CD8^+^ T cells with markers of undifferentiated cells (such as TCF1) and fewer with markers of differentiated cells (TIM3) than tumor tissue. Moreover, blood-derived tumor-specific CD8^+^ T cells had a considerably lower PD1 per cell expression than TILs (Fig. 7b, Supplementary Fig 13b) and also were less apoptosis-prone than tumor-derived tet^+^ CD8^+^ T cells (Fig. 7c and Supplementary Fig. 13c). Relatively high IFNγ and reduced TNF and IL-2 expression is also typical of more advanced dysfunction/exhaustion, whereas IFNγ/TNF/IL-2 co-expression is typical of more undifferentiated states^2^. We found that tumor-specific CD8^+^ TILs produced more IFNγ; blood-derived tumor-specific CD8^+^ T cells produced more TNF and IL-2 after restimulation with PMA and ionomycin (Fig. 7d, e and Supplementary Fig. 13d, e) and contained more poly-functional (IFNγ/TNF/IL-2-coexpressing) cells (Fig. 7e and Supplementary Fig. 13e). Control experiments with single-cell suspensions from blood showed that detection of CD62L and CXCR3 can be somewhat affected by the presence of Liberase (Supplementary Fig. 14), the enzyme used to prepare single-cell suspensions from tumors. Although this effect may be less pronounced when Liberase is added to solid tissue pieces^47^, a caveat seems appropriate concerning the results comparing CD62L and CXCR3 expression between tumor-specific T cells from blood and tumor (Fig. 7a and Supplementary Fig. 13a). The phenotypes determined for tet^+^ cells in peripheral blood and TILs seem to be specific; e.g., most of the tet^+^ cells in peripheral blood were PD1^+^ but tet^-^ CD8^+^ T cells in peripheral blood were generally PD1-negative. Also, tet^+^ TILs showed higher PD1 expression than tet^-^ TILs. TIM3 on tet^+^ TILs was also higher than on tet^-^ TILs (Supplementary Fig. 15).

Additionally, after in vitro stimulation, blood-derived tumor-specific CD8^+^ T cells underwent more rounds of division than did tumor-specific CD8^+^ TILs (Fig. 7f, g). This is consistent with the enhanced apoptosis which we observed for tumor-specific TILs (see Fig. 7c) and with observations that more differentiated exhausted T cells show impaired proliferation and enhanced apoptosis^4,6,42^. Moreover, bulk CD8^+^ T cells from blood also killed autologous tumor cells directly ex vivo (Supplementary Fig. 13f), despite the fact that blood-derived CD8^+^ T cells contained a 15-fold smaller tet^+^ population than CD8^+^ TILs (Fig. 7h and Supplementary Fig. 13g).

### Blood-derived CD8^+^ T cells of triple-treated mice exhibited a significant antitumor effect after adoptive transfer

Finally, we investigated the effect of adoptively transferred CD8^+^ T cells from blood and irradiated tumors of triple-treated mice in tumor-bearing mice conditioned by total body irradiation and treated with IL-2c plus anti-PD1 (Supplementary Fig. 13h). TBI conditioning was used to facilitate the take rate of adoptively transferred cells. Blood-derived CD8^+^ T cells indeed had a significant antitumor effect which was comparable to that of CD8^+^ TILs as shown in Supplementary Fig. 13i for the C51 tumor model. To evaluate how strongly the transferred CD8^+^ T cells proliferate, we co-transferred CFSE-labeled blood-derived and CTV-labeled tumor-derived CD8^+^ cells into C51 tumor-bearing mice. Analysis of single-cell suspensions of recipient blood and spleen 72 h after transfer showed that both proportion (Supplementary Fig. 13j) and number (Supplementary Fig. 13k) of blood-derived donor T cells were higher compared to donor TILs.

To detect transferred T cells at later time points when they could reach the tumor, we used Thy1.1 (recipient) and Thy1.2 (donor) congenic mice bearing a B16-CD133 melanoma tumor. In these experiments, the recipient mice received TBI, anti-PD1, IL-2c and were vaccinated with the M8 peptide + poly(I:C) and anti-CD40 (Fig. 7i). Also in this model, transfer of blood-derived CD8^+^ T cells significantly delayed tumor growth in recipient mice, comparable to transfer of CD8^+^ TILs from irradiated tumors (Fig. 7j). One week after the transfer, more total blood-derived than tumor-derived donor CD8^+^ T cells were detected in TDLNs, blood, tumor, and spleen of the recipient mice (Fig. 7k). Moreover, analysis of tumor-specific CD8^+^ T cells also showed that more blood-derived than tumor-derived donor T cells accumulated after transfer in lymph nodes and tumors of the recipient mice (Fig. 7l). At this time point after the transfer, CXCR3 expression was still high on tet^+^ blood-derived donor CD8^+^ T cells in all analyzed compartments of the recipient mice. The PD1 MFI was significantly upregulated on tumor-infiltrating tet^+^ cells. TIM3^+^ cells tended to increase in tumors but this did not reach statistical significance, perhaps because d7 is an early time point after the transfer. TCF1 stayed high on tet^+^ cells in lymph nodes but decreased in blood and tumor (Supplementary Fig. 16a–c).

These data suggested that blood-derived CD8^+^ T cells from triple-treated mice are a good source for ACT, presumably because they contained a high number of tumor-specific CD8^+^ T cells with a low degree of differentiation associated with high proliferative potential.

## Discussion

Combination of RT with ICB can enhance systemic anti-tumor T cell responses. Here, we show that adding long-acting CD122-directed IL-2c to RT and anti-PD1 can further boost the systemic anti-tumor T cell response. A detailed analysis of different compartments of triple-treated mice demonstrated that tumor-specific CD8^+^ T cells and their stem-like subpopulation most strongly expanded in the blood circulation. In the blood, there also was a more than 150-fold increase in tumor-specific CD8^+^ T cells expressing the migratory chemokine receptor CXCR3. CXCR3 was preferentially expressed on stem-like cells and progressively downregulated on effector-like exhausted and terminally exhausted cells. The amplification of CXCR3^+^ stem-like cells was coupled with a substantial increase in effector-like but not terminally exhausted cells in tumor tissue. Moreover, the abscopal effect in triple-treated mice was dependent on both CD8^+^ and CXCR3^+^ cells. The strong increase in circulating stem-like tumor-specific T cells expressing CXCR3 may therefore explain the improved abscopal effect observed here against an established non-irradiated tumor under triple vs. dual therapies. Consistent with the strong increase in undifferentiated tumor-specific CD8^+^ T cells in blood, we further demonstrate by adoptive transfer experiments that blood after triple treatment represents a rich source of tumor antigen-specific CD8^+^ T cells with high functionality and proliferative capacity for ACT.

So far, little is known about the effects of IL-2 derivatives on tumor antigen-specific T cells and the phenomenon of T cell exhaustion, and the research to date appears controversial. For example, Sahin et al. observed a strong decrease in the expression of PD1, TIM3, and the exhaustion-associated transcription factor TOX in bulk CD8^+^ TILs in a mouse melanoma model upon monotherapy with a long-acting CD122-targeted IL-2 derivative^20^. In contrast, some studies suggest that IL-2 or IL-2 signaling promotes T cell exhaustion^48,49^. At d8 after initiation of therapy, we observed a strong increase in stem-like tumor-specific CD8^+^ T cells in the blood and a substantial increase in effector-like transitory/intermediate cells in the tumor tissue. However, terminally exhausted CD8^+^ T cells were not increased but tended to decrease. Interestingly, we still found stem-like tumor-specific CD8^+^ T cells 2 or 3 weeks after initiation of therapy in all compartments studied here, although the numbers in the TDLNs had decreased at these time points. A recent paper on low-affinity IL-2 fused to an anti-PD1 antibody showed that this IL-2 derivative targeted TILs but not lymph node- or spleen-resident CD8^+^ T cells^50^. This is in line with the higher PD1 expression of TILs compared to extratumoral T cells. In addition, the results of FTY720 blockade experiments suggested that T cell supply from lymph nodes was not required for the observed antitumor effects. Moreover, this IL-2 derivative preferentially induced PD1^+^TIM3^+^ TILs and enhanced their effector functions. Potential effects on T cells in the blood were not examined. While we found the strongest expansion of triple treatment-induced tumor-specific T cells in blood, we also observed substantial expansion of the CD101-negative subset of PD1^+^TIM3^+^ TILs, i.e., of transitory cells which are non-terminally differentiated (effector-like) exhausted cells^5,51^.

Data from both tumor and virus models suggest that PD1/PD-L1 ICB induces preferential expansion of the stem-like subpopulation of exhausted T cells^3,4,6,9^. The anti-PD-L1-induced increase in stem-like cells in chronically LCMV-infected mice has been referred to as a “proliferative burst”^3^. Preferential expansion of stem-like cells, as we also observed with CD122-directed IL-2c, is consistent with a general inverse relationship between differentiation state and proliferative potential.

Our polyfunctional cytokine data for TIL populations with markers of exhausted T cell subpopulations as defined in chronically infected mice (Fig. 4e, f, and Supplementary Fig. 8e, f) showed that the percentage of polyfunctional TILs from triple-treated mice, although higher than in TILs from RT/anti-PD1-treated mice, was still relatively low; moreover, the percentage of polyfunctional CD8^+^ TILs decreased from the stem-like subset to that with markers of the effector-like transitory state to the subset with markers of terminally exhausted cells. Miller et al.^4^ observed a similar trend for SLAM6^+^TIM3^-^ stem-like vs. SLAM6^-^TIM3^+^ CD8^+^ TILs for IFNγ/TNF double-positive TILs. The proportion of polyfunctional IFNγ/TNF double-positive CD8^+^ TILs which we found is considerably lower than described for non-exhausted T cells, e.g., in a model of autoimmune diabetes where approx. 70% of lymph node or pancreatic antigen-specific CD8^+^ T cells were double-positive for IFNγ and TNF^52^ or compared with what we found for a peptide-specific CD8^+^ T cell line induced in vitro, using peptide-pulsed splenocytes and restimulated weekly. Our data are thus consistent with at least partial exhaustion or dysfunction^42^. Nevertheless, it is likely that the addition of CD122-targeted IL-2 affects gene expression of the tumor-specific T cell subsets studied here. This could contribute to the quantitative changes among the subsets as well as the enhanced effector functions/polyfunctionality which we observed. Whether such likely changes in gene expression only quantitatively and qualitatively alter the known subsets of the exhaustion lineage or (also) give rise to distinct (as yet unknown) differentiation states would need to be clarified in future studies. Very recently, it has been shown that adding rIL-2 to anti-PDL1 in chronic LCMV infection modifies the CD8^+^ T cell exhaustion program in a way that non-exhausted “better effectors” are induced^53^. However, this was dependent on binding of rIL-2 to CD25 and was not observed with a mutant CD122-targeted IL-2 molecule. “Better effectors” were also found with CD122-targeted IL-2 fused to anti-PD1 but not with free CD122-targeted IL-2 combined with anti-PD1^54^.

TDLNs appear to be crucial for RT-mediated^33,34^ and ICB-mediated^55^ antitumor responses, and have recently been identified as important reservoir for stem-like tumor-specific CD8^+^ T cells^56,57^. The following of our observations support the notion that stem-like CD8^+^ T cells from TDLNs are important for RT/anti-PD1/IL-2c-induced antitumor effects: (i) after co-administration of FTY720, stem-like tumor-specific T cells accumulated in lymph nodes draining the irradiated tumor; (ii) concomitantly, more differentiated exhausted TIM3^+^TCF1^-^PD1^+^ cells decreased in tumor tissue after co-administration of FTY720 and tumor control was compromised; (iii) CXCR3^+^ stem-like tumor-specific CD8^+^ T cells in the blood circulation expanded strongly; (iv) the abscopal effect under triple therapy depended on the CXCR3/CXCL9/10/11 interaction. The conclusion that stem-like CD8^+^ T cells from TDLNs are important for the RT/anti-PD1/IL-2c-induced abscopal effect is consistent with observations by Buchwald et al.^34^. They observed that the mild abscopal effect induced by RT monotherapy was completely abrogated when the lymph node draining the irradiated tumor was included in the irradiation field and the number of stem-like CD8^+^ T cells concomitantly dropped both at the irradiated and the non-irradiated sites.

In a recent paper, Pimentel et al., using mice with a single tumor, found a better effect for triple treatment with RT, tumor neovasculature-targeting L19-IL-2, and anti-PD1 compared to RT/anti-PD1 or RT/L19-IL-2 in the LLC model; in the CT26 and C51 colon carcinoma models the differences were significant only compared to RT/anti-PD1^29^. Also, Pieper et al., studying responses of an irradiated tumor and associated lung micrometastases in a head and neck cancer model, found a superior antitumor effect of RT/pegylated IL-2/anti-PD-L1 triple treatment and better survival compared to RT/anti-PD-L1 but not RT/pegylated IL-2^30^. In our single-tumor experiments, triple therapy controlled the irradiated tumor better than RT/anti-PD1 in both the B16-CD133 and C51 models, whereas it was better than RT/IL-2c only in the B16-CD133 but not the C51 model. The latter observation is similar to the findings in the C51 one-tumor model by Pimentel et al. mentioned above. We describe here abscopal responses of established non-irradiated tumors to triple treatment with RT, ICB, and an IL-2 derivative. In the two different bilateral-tumor models which we have studied, triple treatment induced better abscopal responses against the non-irradiated tumor than all double treatments.

A relatively large number of IL-2 formulations and derivatives are in clinical evaluation in phase 1–3 trials as monotherapy or in combination with other treatments. Regarding preferential receptor targeting, unbiased, CD122-biased, CD25-biased, and CD25/CD122-biased formulations are being evaluated. Regarding cell targets, these are either untargeted, tumor cell-targeted, immune cell-targeted, or TME-targeted^58^. However, late-stage trials of CD122-biased pegylated IL-2 and nivolumab have recently turned out disappointing^59^. A phase II trial of high-dose rIL-2 with stereotactic body RT also fell short of expectations (NCT01416831)^60^. Combinations of an IL-2 derivative with RT ± ICB are also under clinical evaluation (NCT03705403)^61^. The current status of clinical testing indicates that in-depth preclinical investigations of IL-2-based drugs alone and in combination with other treatments are extremely important for further clinical development, both with regard to anti-tumor effects and with regard to mechanistic aspects.

It has been recognized a while ago that CD8^+^ T cells with a stem-like phenotype (e.g., CD44^low^CD62L^high^ T memory stem cells^62^, progenitor exhausted SLAMF6^+^TIM3^−4^ or CD69^-^CD39^-^ TILs^63^) are beneficial for ACT, in large part due to the high proliferative capacity of these relatively undifferentiated cells. Our findings support this notion. Young T cells with stem-like phenotype are present only at low frequencies in tumors and the large majority of TILs from untreated tumors are usually terminally differentiated/exhausted^4,6^. Blood has been recognized as an alternative source for tumor-specific T cells for adoptive transfers^35,64–67^. However, in most of these studies, donor T cells were isolated from blood of untreated patients. Interestingly, in order to increase circulating tumor-specific T cells for generating T cell infusion products, Kandalaft et al. vaccinated ovarian cancer patients before apheresis with a personalized dendritic cell vaccine^64^. We demonstrate here in mice that a triple combination of a CD122-directed IL-2 derivative, RT, and anti-PD1 may act as a rather potent personalized in situ vaccine and tremendously increase the number of tumor-specific CD8^+^ T cells in the blood circulation. Despite an approx. 15-fold lower percentage of tumor-specific cells, blood-derived CD8^+^ T cells were similarly effective in adoptive transfer experiments as CD8^+^ TILs from irradiated tumors. This relatively high efficacy of the blood-derived CD8^+^ T cells was associated with characteristics of undifferentiated cells in the tumor-specific population such as a much higher percentage of TCF1^+^ cells, a much lower PD1 MFI, a much lower percentage of TIM3^+^ cells, a lower apoptosis propensity and a higher proliferative capacity and polyfunctionality than tumor-specific CD8^+^ TILs. Moreover, donor TILs from triple-treated mice have relatively good effector functions, whereas it is conceivable that blood-derived donor T cells mainly acquire effector functions only after immigration into the tumor tissue of recipient mice, which may take some time^68^.

In summary, we show that triple treatment with RT/anti-PD1/IL-2c greatly increased the number of tumor-specific CD8^+^ T cells compared with dual RT/anti-PD1 treatment. The highest increase (>50-fold) was found in the blood circulation. Stem-like tumor-specific CD8^+^ T cells expanded mostly in the peripheral blood; these highly expanded cells expressed CXCR3. In tumor tissue, effector-like, but not terminally differentiated, exhausted T cells increased. Consistent with the large increase in undifferentiated tumor-specific CD8^+^ T cells with high proliferative capacity in the blood circulation, we observed a CD8- and CXCR3-dependent enhancement of the RT-induced abscopal effect against a distant, non-irradiated tumor and found that blood-derived CD8^+^ T cells generated by such a triple treatment can be a rich source of tumor antigen-specific CD8^+^ T cells for ACT.

## Methods

### Mice and cell lines

All animal experiments were performed in accordance with the German Animal License Regulations and were approved by the animal care committee of the Regierungspräsidium (Federal Ministry for Nature, Environment and Consumers’ Protection) Freiburg, Freiburg, Germany (registration numbers: G18/066, G20/016). C57BL/6Nrj and BALB/c mice were purchased from Janvier Labs. C57BL6-Thy1.1 mice were from the local stock of the SPF animal facility of the University of Freiburg. Mice were used at the age of 8–12 weeks at the start of the experiment. Animals were housed under specific pathogen-free conditions with 12 hours light/dark cycle, 21–25 °C, 45–65% humidity. A maximum of five mice per cage were housed with unlimited access to food and water. Mice were euthanized by CO_2_ asphyxiation followed by cervical dislocation when the pre-specified conditions for termination of animal experiments according to the German Animal License Regulations were reached.

B16-CD133 melanoma cells were generated as described before^69^. The C51 colon carcinoma cell line was obtained from Dr. Mario Paolo Colombo (Milan). B16-CD133 tumor cells and C51 tumor cells were cultured in complete RPMI-1640 medium (Gibco) and complete DMEM medium (Gibco), respectively, each containing 10% FBS (Gibco) and 100 IU/ml penicillin-streptomycin (Gibco). All in vitro T cell assays were performed using complete RPMI-1640 medium containing 10% FBS (Gibco), 100 IU/ml Penicillin-Streptomycin (Gibco), 10 mM HEPES (Sigma-Aldrich), 1x NEAA (Gibco), 2 mM L-Glutamine (Gibco), and 50 mM β-mercaptoethanol (Sigma-Aldrich).

### Tumor models and treatment

Cells resuspended in PBS were mixed 1:1 with Matrigel (Corning) and 5 × 10^5^ C51 or 2 × 10^5^ B16-CD133 cells were inoculated subcutaneously (s.c.) into the right flank of the mice. In the one-tumor model, mice were randomized for treatment according to tumor size approx. 12 and 16 days after tumor inoculation for the B16-CD133 and the C51 tumor model, respectively. For abscopal tumor models, 2 × 10^5^ cells were additionally injected into the left flank 6 days (B16-CD133 model) or 10 days (C51 model) later. Mice were randomized for treatment according to tumor size 4-6 days after secondary tumor implantation. Tumors were irradiated locally with two fractions of 12 Gy (B16-CD133 model) or 8 Gy (C51 model) on consecutive days, followed by intraperitoneal (i.p.) injection of 200 μg anti-PD1 antibody (RMP1-14, BioXCell, BE0146) and five or four daily i.p. injections of 9 μg IL-2c from day 3 after treatment start. For IL-2c preparation, we mixed mouse rIL-2 (ImmunoTools, 12340028) and the monoclonal S4B6 antibody which we purified from culture supernatants of the S4B6-1 hybridoma (ATCC clone HB-10968, RRID:CVCL_9236) at a molar ratio of 2: 1. We characterized the complexes by gel filtration which showed a complete shift in the elution profile of IL-2c compared with the uncomplexed S4B6 antibody^70^.

Tumor irradiation was performed using an RS2000 X-ray unit (RadSource) operated at 160 kV with a 0,3 mm cooper filter delivering a 3,744 Gy/min. Mice were anesthetized using i.p. injections of ketamin (10%; 80 mg/kg) and medetomidin (1 mg/ml; 0.8 mg/kg), and eyes were covered with Bepanthen cream (ophthalmic ointment). Mice were positioned in a custom-made plastic jig with a size-adjustable aperture through which the tumor was pulled into the irradiation window; the rest of the mouse body was fully shielded with lead (Supplementary Fig. 1a). To ensure uniformal dose delivery, mice were turned 180^o^ for the second irradiation. Dosimetric measurements with phantoms using the TLD method showed that at a distance of 5 mm, only 0.06% of the dose delivered to the tumor is detectable. To wake the mice up after the irradiation, 35 µl atipamezole (5 mg/ml; 7 mg/kg) was injected.

To block T cell egress from lymph nodes, FTY720 (25 μg per mouse Fingolimod, Cayman Chemical, 10006292) was i.p. injected 1 day before treatment start and then every other day to maintain the blockade. CD8^+^ T cells were depleted by injecting 200 μg/mouse of anti-CD8β antibodies (YTS 156.7.7) i.p. as indicated in Fig. 5a. To block CXCR3/CXCL9/10/11, anti-CXCR3 antibody (CXCR3-173, BioXCell, BE0249) was injected i.p. as indicated in Fig. 6a, using 100 and 400 μg/mouse before and after starting the IL-2 injections, respectively. Tumor growth was calculated as length×width×height measured by caliper. Survival was defined as the time point after treatment start when either the primary or the secondary tumor had reached a size of 2,000 mm^3^.

### Preparation of single-cell suspensions and determination of absolute cell numbers

For single-cell suspension preparation, tumors were weighed and digested in 5 ml of PBS plus MgCl_2_ plus CaCl_2_ (Gibco; Thermo Fisher Scientific) supplemented with 120 μg/ml of Liberase and 50 μg/ml of DNAse for 20 min at 37 °C while rotating. After the incubation, tumor pieces were mechanically ground through a 70-μm sieve and filtrated through 30-μm cell strainers (Miltenyi Biotec). The spleens and lymph nodes were squeezed through a 70-μm strainer. Red blood cell lysis was performed using 1X red blood cell lysis buffer (eBioscience). After the preparation of single-cell suspensions, leukocytes were counted based on size and morphology using trypan blue staining, and the frequency of each population among CD45^+^ cells was determined by flow cytometry thereafter. To determine the absolute number of cells of a certain population, the percentage among CD45^+^ cells was multiplied with the total number of counted viable leukocytes. In tumor samples, the number of cells of a specific population was calculated per gram of tumor. In peripheral blood, the number of cells of a specific population was calculated per ml of blood. For secondary lymphoid organs, we determined absolute numbers for total spleen and tumor-draining lymph nodes (inguinal + axillary), respectively.

### Flow cytometry analysis

Single-cell suspensions were incubated with ZombieRed (BioLegend, 423110) or ZombieNIR (BioLegend, 423106) to exclude dead cells, then with rat anti-mouse CD16/32 Fc receptor–blocking antibody (clone 2.4G2). When propidium iodide (PI; Miltenyi Biotec, 130-093-233) was used for T cell exclusion, it was added to the samples directly before measurement. The M8 tetramer-PE (H-2K^b^, MuLV p15E, KSPWFTTL; 1:200) and the AH1 tetramer-PE (H-2L^d^, MuLV gp70, SPSYVYHQF; 1:200) both from Baylor College of Medicine (Houston, TX) with CD8-AF700 (KT15, Bio-Rad; 1:50) were used to detect tumor-specific CD8^+^ T cells. The OVA tetramer-APC (H-2K^b^, SIINFEKL; 1:200) and the Ag85A tetramer-APC (H-2L^d^, Mtb, MPVGGQSSF; 1:200) tetramers were used as specificity control (see Supplementary Fig. 3a). The following anti-mouse antibodies were purchased from BioLegend: CD45-BV510 (30-F11; 1:200), CD3-PerCP-Cy5.5 (145-2C11; 1:200), PD1-BV421 and FITC (29 F.1A12; 1:200), TIM3-BV605 (RMT3-23; 1:200), CD101-PE-Cy7 (Moushi101; 1:200), CD69-FITC (H1.2F3; 1:200), CD28-PE-Cy7 (37.51; 1:200), CXCR3-APC-Fire750 (CXCR3-173; 1:200), CD62L-APC-Cy7 (MEL-14; 1:200), TNF-APC-Cy7 (MP6-XT22; 1:200), CD107a-BV421 (1D4B; 1:200), Ki67-BV605 (16A8; 1:200), IL-2-APC and BV510 (JES6-5H4; 1:200), CD49b-FITC (DX5; 1:200), F4/80-BV421 (BM8; 1:200), CD11b-PE-Cy7 (M1/70; 1:200), Ly6C-PE and BV605 (HK1.4; 1:200), Thy1.1-PerCP (OX-7; 1:200), Thy1.2-PerCP-Cy5.5 or APC (53-2.1; 1:200); from eBiosciences/Invitrogen: CD3-APC-eFluor780 (145-2C11; 1:200), CD122-FITC (TM-b1; 1:200), T-bet-eFluor660 (4B10; 1:200), IFNγ-FITC (XMG1.2; 1:200), TOX-APC (TXRX10; 1:200), GR1-APC (RB6-8C5; 1:200); from BD Biosciences: CD11c-BV650 (HL3; 1:200), CD103-PE-CF594 (M290; 1:200), Ly6G-PE (1A8; 1:200); from Miltenyi Biotec: CXCR3-APC (REA724; 1:50), TIM3-APC (REA602; 1:50); or from Cell Signaling: TCF1-Alexa Fluor 647 and PacificBlue (C63D9; 1:200).

When needed, cells were fixed with the Foxp3/Transcription Factor Staining Buffer Set (eBiosciences, 00-5523-00) or Intracellular Staining Kit (eBiosciences, 88-8824-00) according to the manufacturer’s instructions. The APC Annexin V/PI Kit (BioLegend, 640920) was used to determine the proportion of apoptotic cells. Data were acquired using a CytoFlex S Flow Cytometer (Beckman Coulter) with CytExpert 2.4.0.28 software followed by data analysis with FlowJo 10.4 software.

### In vitro conversion to CD101^+^ cells

CD8^+^ T cells were isolated from the tumor or blood single-cell suspensions of C51 tumor-bearing mice treated with RT/anti-PD1/IL-2c on day 8 after treatment start. Specific subsets were flow-cytometrically sorted (MoFlo Astrios EQ with Summit v 6.3.1. software) using the following gating strategies: live ZombieRed^-^CD45^+^CD8^+^PD1^+^TIM3^+^CD101^-^ (from the tumor) and live ZombieRed^-^CD45^+^CD8^+^PD1^+^TIM3^-^CD101^-^ (from the peripheral blood). The sorted CD8^+^ T cells were then co-cultured with IFNγ-pretreated C51 tumor cells at a ratio of 1:5 in presence of 10 IU/mL rmIL-2. After 3 days, the cells were assayed for expression of TIM3 and CD101.

### Ex vivo flow cytometric analysis of CXCL9/CXCL10-secreting cell subsets

Mice were injected i.v. with 0.25 mg of BrefA (Sigma-Aldrich, B6542-5MG) and sacrificed 12 h later. Tumor single-cell suspensions, prepared as described above, were stained with ZombieRed, followed by surface antibody staining. Cells were fixed, permeabilized, and stained with CXCL9-eFluor660 (MIG-2F5.5; 1:100) or Armenian Hamster IgG Isotype Control-eFluor660 (eBio299Arm; 1:100). For CXCL10 staining, unconjugated CXCL10 antibodies (polyclonal, 0.2 mg/ml; 1:25) or goat IgG isotype (polyclonal, 0.2 mg/ml; 1:25) from R&D systems were used followed by staining with secondary antibodies (Rabbit anti-Goat AF488, Invitrogen; 1:100).

### Ex vivo T cell re-stimulation

To evaluate CD8^+^ T cell effector functions, TILs were re-stimulated in vitro for 1 h at 37 ^o^C, 5% CO_2_ in RPMI medium with 1 μg/ml AH1 peptide (SPSYVYHQF, AnaSpec Inc.) and CD107a antibodies; 1x BrefA (BioLegend, 420601) was added for additional 4 h. Alternatively, TILs or blood-derived cells were stimulated with PMA (50 ng/ml) plus ionomycin (1 µg/ml) plus 1x BrefA for 4 h. Thereafter, intracellular cytokine staining was performed for flow cytometry analysis.

### In vitro induction of M8-specific CD8^+^ T cells

To induce M8-specific T cells in vitro, 4×10^5^ naive splenocytes were plated as responder cells per well into 96-well plates. Stimulatory splenocytes were incubated with 1 µM of M8 peptide (KSPWFTTL, GenScript) for 2 h and irradiated thereafter with 30 Gy; 3×10^5^ cells were then added to the wells. The cell medium (see above) was supplemented with 10 IU/mL of rmIL-2 and half of the medium was refreshed 3 times per week. Cells were restimulated with peptide-pulsed irradiated splenocytes every week and M8-specific CD8^+^ T cells were used for the assay 4 weeks after the start of the experiment.

### Analysis of gene expression and survival in TCGA datasets

Analysis of patients´ data was performed using GEPIA2.0 (http://gepia2.cancer-pku.cn/) based on TCGA SKCM cohort. Database was accessed on 13 November 2019. Correlation analysis between *CXCR3* and *IL2RB* genes was performed using Pearson correlation coefficient. Patient overall survival was evaluated by Kaplan-Meier survival analysis with low or high expression of CXCR3 defined by median expression (50% cut-off).

### In vitro cytotoxicity

To compare the cytotoxicity of blood- and tumor-derived CD8^+^ T cells, target cells were labeled with the PKH26 Red Fluorescent Cell Kit (Sigma-Aldrich, PKH26GL-1KT) and 5 × 10^3^ C51 target tumor cells were incubated with isolated CD8^+^ T cells at a ratio of 1:1, 1:7,5, and 1:15 for 48 h. Cell death of PKH26^+^ tumor cells was assessed by flow cytometry using 7-AAD (eBioscience, 00-6993-50). Labeled target cells incubated without T cells were used as controls. Specific cytotoxicity was determined as (% 7-AAD^+^ of PKH26^+^ target cells)—(% 7-AAD^+^ of PKH26^+^ control cells).

### Ex vivo proliferation assay

CD8^+^ cells were isolated from blood or irradiated tumors, labeled with CFSE (Invitrogen, C34554) according to the manufacturer’s protocol. Cells were plated at 50,000 cells/200 μl in 96-well U-bottom plates coated with 1 μg/ml anti-CD3 (Invitrogen) in RPMI with 1 μg/ml anti-CD28 (Invitrogen), and 50 IU/mL rmIL-2 (ImmunoTools). After 72 h, cells were harvested and stained for flow cytometry. Proliferation was analyzed using FlowJo’s proliferation platform.

### In vivo transfer

Donor mice were treated with RT/anti-PD1/IL-2c and single-cell suspensions were prepared from blood or irradiated tumors at day 8 after treatment start. CD8^+^ cells from blood were isolated using a CD8^+^ T Cell Isolation kit (MiltenyiBiotec, 130-104-075), and TILs were isolated using CD8 (TIL) MicroBeads (MiltenyiBiotec, 130-116-478). Recipient mice were inoculated with tumor cells (5 × 10^5^ and 2 × 10^5^ C51 and B16-CD133, respectively) 7 days before and received 4 Gy total body irradiation (TBI) 24 h before ACT. C51 tumor-bearing recipient mice received 2 × 10^6^ isolated CD8^+^ T cells i.v. and at the same day 200 μg anti-PD1 as well as 3 × 9 μg IL-2c on days 1, 2, and 3; this combination of anti-PD1 and IL-2c was injected for 2 more weeks. In some experiments, donor blood-derived CD8^+^ cells were labeled with CFSE (Invitrogen, C34554) and tumor-derived CD8^+^ cells were labeled with CTV (Invitrogen, C34557) before the transfer. T cell proliferation in recipients’ organs was analyzed by flow cytometry 3 days after the transfer.

C57BL6-Thy1.1 mice received 2 × 10^6^ CD8^+^ donor T cells i.v. together with anti-PD1 and IL-2c as described above. Additionally, these mice were s.c. immunized with 50 μg M8 peptide (KSPWFTTL, GenScript), 50 μg poly(I:C), and 50 μg anti-CD40 antibodies (MR-1, BioXCell, BE0017-1). T cell proliferation was analyzed by flow cytometry 7 days later. The control groups of recipient mice received all described treatments except the transferred donor T cells.

### Statistical analysis

Results are presented as mean ± SEM. For two-group comparisons, statistical analysis was performed using a paired or unpaired two-tailed Student’s *t* test. For multiple comparisons, one-way ANOVA was used followed by the “Dunnet” or “Tukey” correction. Survival data were compared using the log-rank Mantel-Cox test. *P* < 0.05 was considered significant. Statistical analyses were performed using Prism version 8.0 (GraphPad).

### Reporting summary

Further information on research design is available in the Nature Portfolio Reporting Summary linked to this article.

### Supplementary information

Supplementary Information

Reporting Summary

### Source data

Source Data

## Data Availability

Source data are provided with this paper, except for Supplementary Fig. 9c–e. For Supplementary Fig. 9c, publicly available datasets were used from Miller et al.^4^—accessible through the GEO series accession number GSE122713; or Hudson et al.^5^—available from the NCBI Sequence Read Archive under BioProject PRJNA497086 and normalized gene counts are from Supplementary Information of the paper^5^. The publicly available online tool GEPIA2.0 utilized to generate the graphs for Supplementary Fig. 9d, e (http://gepia2.cancer-pku.cn, as indicated in methods) does not make the data available. All other data are available in the article and its Supplementary files or from the corresponding author upon request. Source data are provided with this paper.
