# Author Correction: Expansion of circulating stem-like CD8^+^ T cells by adding CD122-directed IL-2 complexes to radiation and anti-PD1 therapies in mice

**DOI:** 10.1038/s41467-024-50995-6

**Published:** 2024-08-05

**Authors:** Kateryna Onyshchenko, Ren Luo, Elena Guffart, Simone Gaedicke, Anca-Ligia Grosu, Elke Firat, Gabriele Niedermann

**Affiliations:** 1 Department of Radiation Oncology, Faculty of Medicine, University of Freiburg, Freiburg, Germany; 2 Faculty of Biology, University of Freiburg, Freiburg, Germany; 3 Laboratory of Biosynthesis of Nucleic Acids, Institute of Molecular Biology and Genetics of NASU, Kyiv, Ukraine; 4 German Cancer Consortium (DKTK), Partner Site Freiburg, Freiburg, Germany; 5 German Cancer Research Center (DKFZ), Heidelberg, Germany; 6 Division of Thoracic Tumor Multimodality Treatment, Cancer Center, West China Hospital, Sichuan University, Chengdu, Sichuan China

Correction to: *Nature Communications* 10.1038/s41467-023-37825-x, published online 12 April 2023

In the published version of the manuscript, Figure 2e contained an error, in which one flow cytometry plot for ‘Tumor’ was inadvertently shown twice in column 2 and 3. This has now been corrected in both the online and PDF version.

