# Supplementary material for: Expansion of circulating stem-like CD8+ T cells by adding CD122-directed IL-2 complexes to radiation and anti-PD1 therapies in mice
Source: Nat Commun. 2023 Apr 12;14:2087. doi: 10.1038/s41467-023-37825-x (PMC10097749; doi:10.1038/s41467-023-37825-x)
Supplement: Supplementary file 1 — Supplementary Information [file 41467_2023_37825_MOESM1_ESM.pdf]

Supplementary Information

Expansion of circulating stem-like CD8$^+$ T cells by adding CD122-directed IL-2 complexes to radiation and anti-PD1 therapies

Kateryna Onyshchenko[1,2,3,4,5], Ren Luo[1,2,4,5,6], Elena Guffart[1,2], Simone Gaedicke[1], Anca-Ligia Grosu[1,4,5], Elke Firat[1], and Gabriele Niedermann[1,4,5]*

[1]Department of Radiation Oncology, Faculty of Medicine, University of Freiburg, Freiburg, Germany.

[2]Faculty of Biology, University of Freiburg, Freiburg, Germany.

[3]Laboratory of Biosynthesis of Nucleic Acids, Institute of Molecular Biology and Genetics of NASU, Kyiv, Ukraine

[4]German Cancer Consortium (DKTK), Partner Site Freiburg, Freiburg, Germany.

[5]German Cancer Research Center (DKFZ), Heidelberg, Germany

[6]Thoracic Oncology Ward, Cancer Center, West China Hospital, Sichuan University, Chengdu, China

*Corresponding author: Gabriele Niedermann, Department of Radiation Oncology, University of Freiburg, Robert-Koch-Strasse 3, 79106 Freiburg, Germany

Tel: +49-76127095140; E-mail: gabriele.niedermann@uniklinik-freiburg.de

Supplementary Figure 1

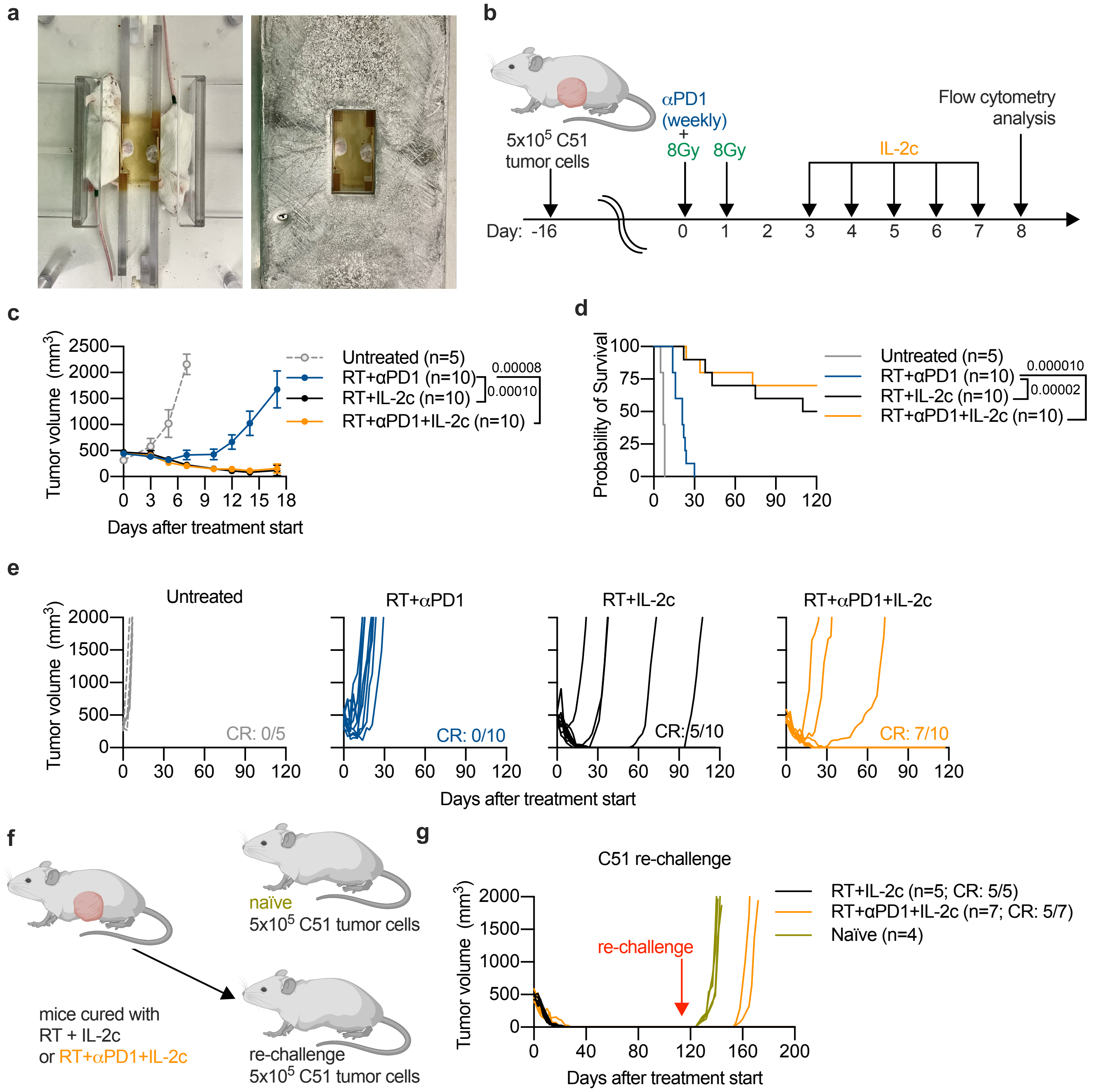

Supplementary Figure 1. Adding IL-2c to RT + αPD1 improves therapeutic efficacy in the C51 colon carcinoma model. **a**, Example of mouse fixation for RT; the tumor to be irradiated was pulled into the irradiation window of the plastic jig (left), the rest of the animal was covered by lead shielding (right). Thermoluminescence dosimetry (TLD) measurements showed that at a distance of 5 mm only 0.06% of the dose delivered to the primary tumor is detectable. **b**, Scheme for treatments and T cell analyses. Tumor growth (**c**), and survival of mice (**d**) (n = 5–10 mice per group). **e**, Individual tumor growth curves of C51 tumors (CR, complete response; indicates how many mice were cured). The whole experiment was repeated once with the same number of mice per group, with similar results. Error bars in (c) represent SEM. **f**, Scheme of tumor re-challenge experiment using the mice with complete tumor remission from the experiment shown in panels c-e. **g**, Individual tumor growth curves for naive and cured mice challenged with $5x10^5$ C51 tumor cells. *P* values are shown; statistical comparisons were performed using one-way ANOVA with Tukey's multiple comparisons test (c), or log-rank test (d). Source data are provided as a Source Data file.

Supplementary Figure 2

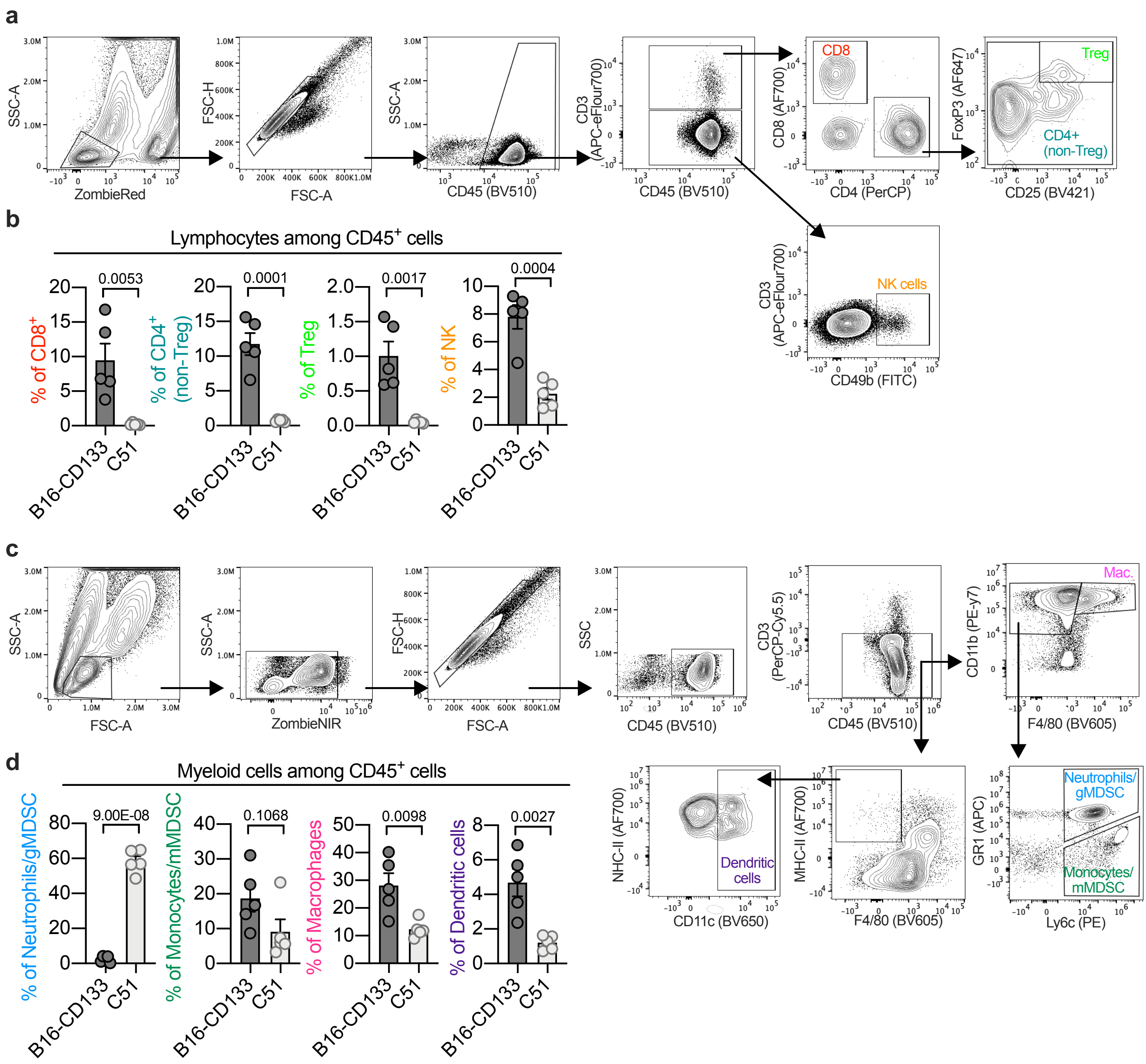

Supplementary Figure 2. Comparison of tumor immune profiles between B16-CD133 melanoma and C51 colon carcinoma model. a, c. Gating strategy used to characterize tumor-infiltrating lymphocyte (**a**) and myeloid cell (**c**) subpopulations. **b, d,** Frequency of various lymphoid (**b**) and myeloid (**d**) immune cells among CD45^{+} cells in B16-CD133 (gray) and C51 (white) tumor models (n = 5 individual mice). Data are presented as mean ± SEM and were collected from two independent experiments with 2–3 mice for each group. *P* values are shown; statistical comparisons were performed using two-tailed unpaired Student's t-test. Source data are provided as a Source Data file.

Supplementary Figure 3

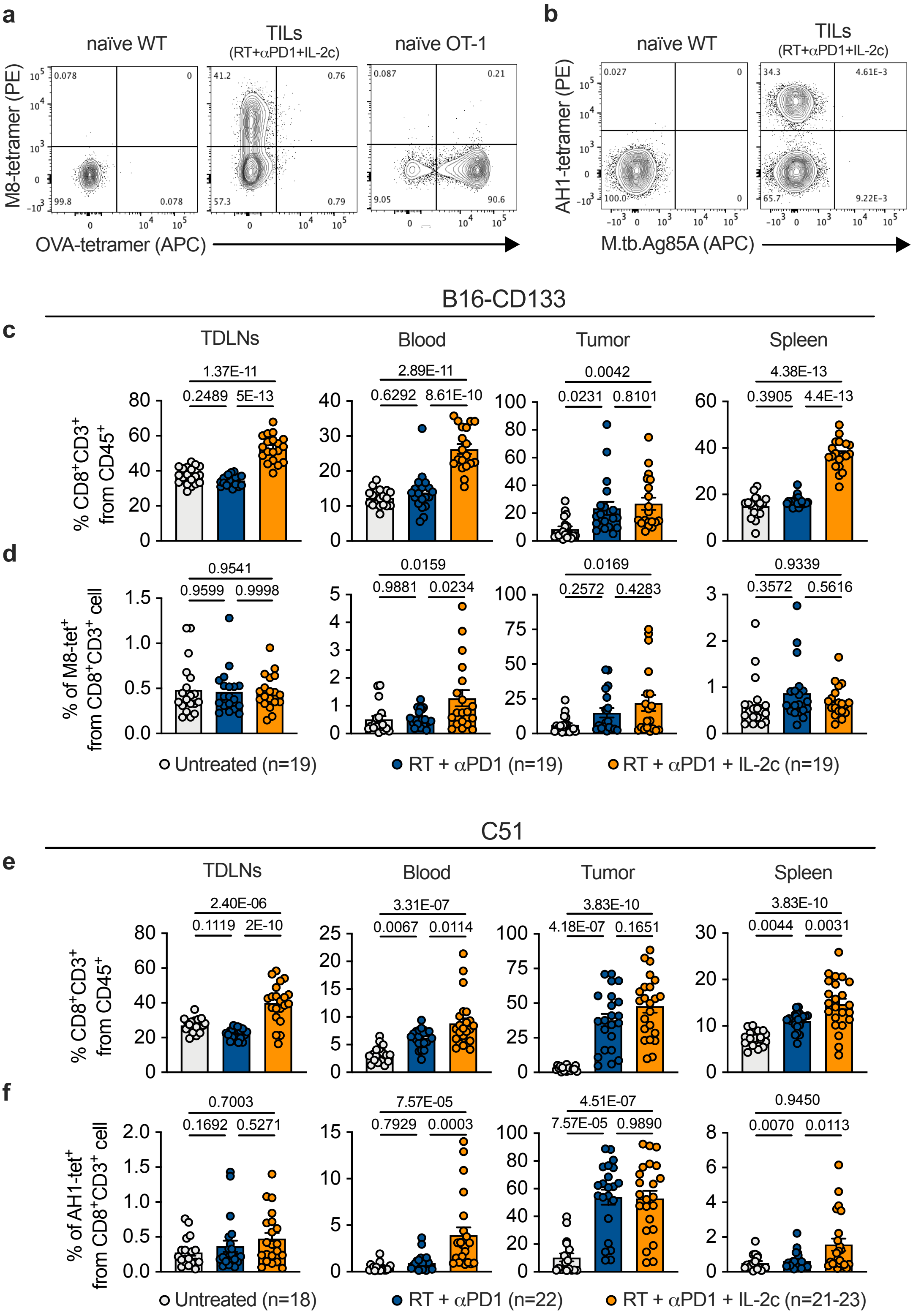

Supplementary Figure 3. Percentage of CD8$^+$ and tumor-specific CD8$^+$ T cells in different compartments of tumor-bearing mice. a-b, Flow cytometry examples of tetramer specificity; **a,** Naïve wild-type splenocytes, OT-I splenocytes or TILs from RT + αPD1 + IL-2c-treated B16-CD133 tumor-bearing mice were stained with H-2K^b/M8 (PE-labeled) and H-2K^b/OVA (APC-labeled) tetramers; **b,** naïve wild-type splenocytes or TILs from RT + αPD1 + IL-2c-treated C51 tumor-bearing mice were stained with H2-L^d/AH1 (PE-labeled) and H2-L^d/Ag85 (APC-labeled) tetramers. **c-f**, Percentage of CD8$^+$ among CD45$^+$ (**c, e**) and tetramer$^+$ among CD8$^+$CD3$^+$ (**d, f**) cells in TDLNs, blood, tumor and spleen from indicated treatment groups for B16-CD133 (n = 19) (**c, d**) and C51 (n = 18–23) (**e, f**) tumor models. Data are presented as mean ± SEM and were collected from at least six independent experiments with 2–4 mice in each treatment group. *P* values are shown; statistical comparisons were performed using one-way ANOVA with Tukey's multiple comparisons test. Source data are provided as a Source Data file.

Supplementary Figure 4

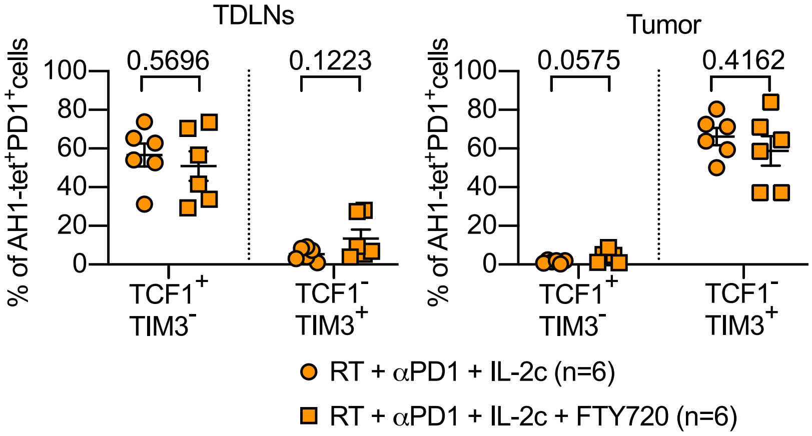

Supplementary Figure 4. Effect of FTY720 on T cell phenotype. a, Percentage of $TCF1^{+}TIM3^{-}$ and $TCF1^{-}TIM3^{+}$ cells in TDLNs (left) and tumor (right) of C51 tumor-bearing mice treated with RT + αPD1 + IL-2c with (squares) or without (circles) FTY720 (n = 6 individual mice). *P* values are shown and error bars indicate mean ± SEM; statistical comparisons were performed using two-tailed unpaired Student's t-test. Source data are provided as a Source Data file.

Supplementary Figure 5

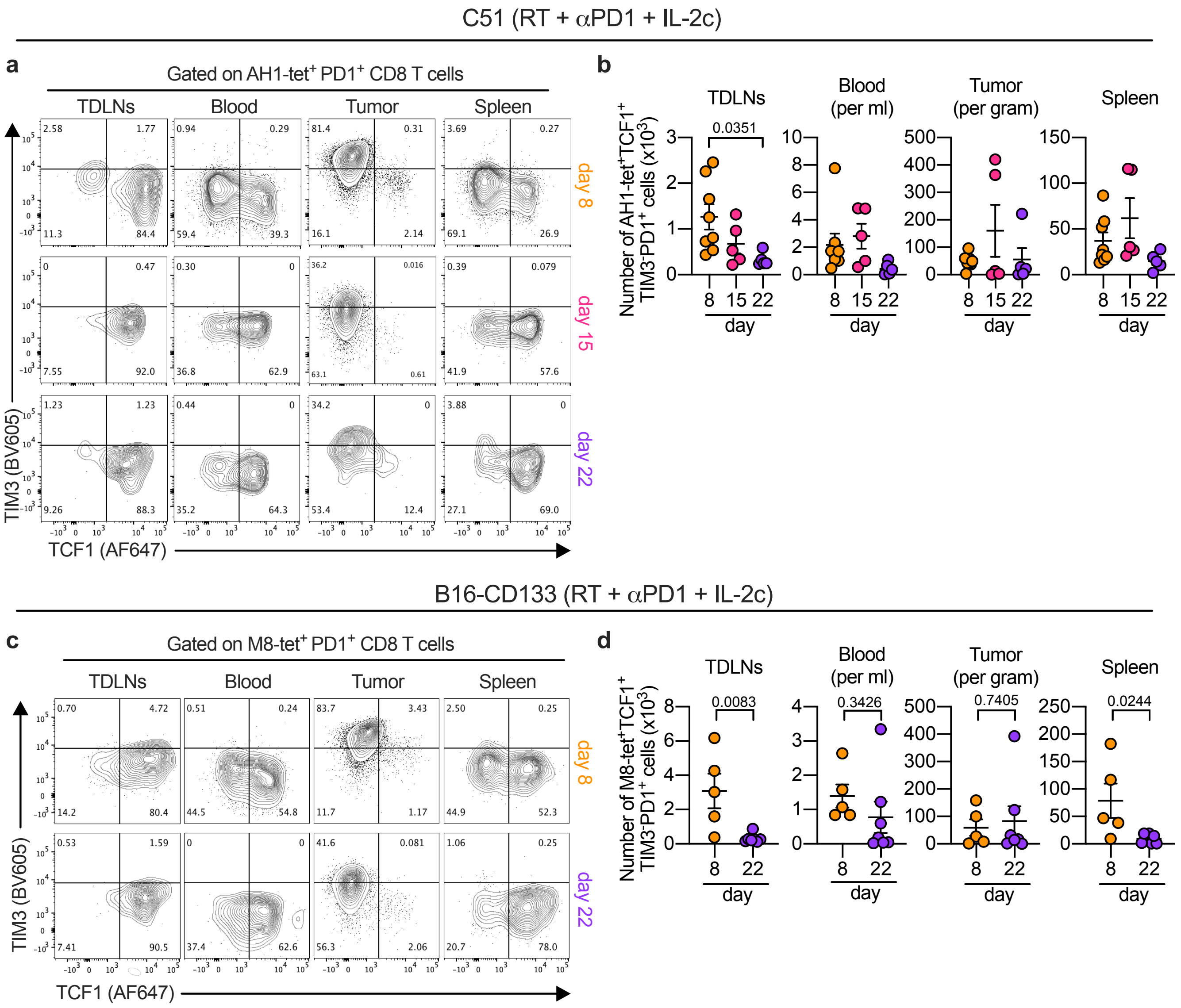

Supplementary Figure 5. Long-term persistence of TCF1⁺TIM3⁺PD1⁺ "stem-like" CD8⁺ T cells.

a, c, Flow plot examples for TIM3/TCF1 expression among AH1-tet$^+$PD1$^+$ (**a**) and M8-tet$^+$PD1$^+$ (**c**) cells in indicated compartments of RT + αPD1 + IL-2c-treated mice at different time points after treatment start. **b, d,** Numbers of TCF1$^+$TIM3$^-$ PD1$^+$ AH1-tet$^+$ (b) and M8-tet$^+$ (d) CD8$^+$ T cells at d8 (C51, n = 8; B16-CD133, n = 5), d15 (C51, n = 5), and d22 (C51, n = 5; B16-CD133, n = 7) after treatment with RT + αPD1 + IL-2c. Data are presented as mean ± SEM and were collected from 2–3 independent experiments for each group. Symbols represent individual mice. *P* values are shown; statistical comparisons were performed using one-way ANOVA with Tukey's multiple comparisons test (b) or two-tailed unpaired Student's t-test (d). Source data are provided as a Source Data file.

Supplementary Figure 6

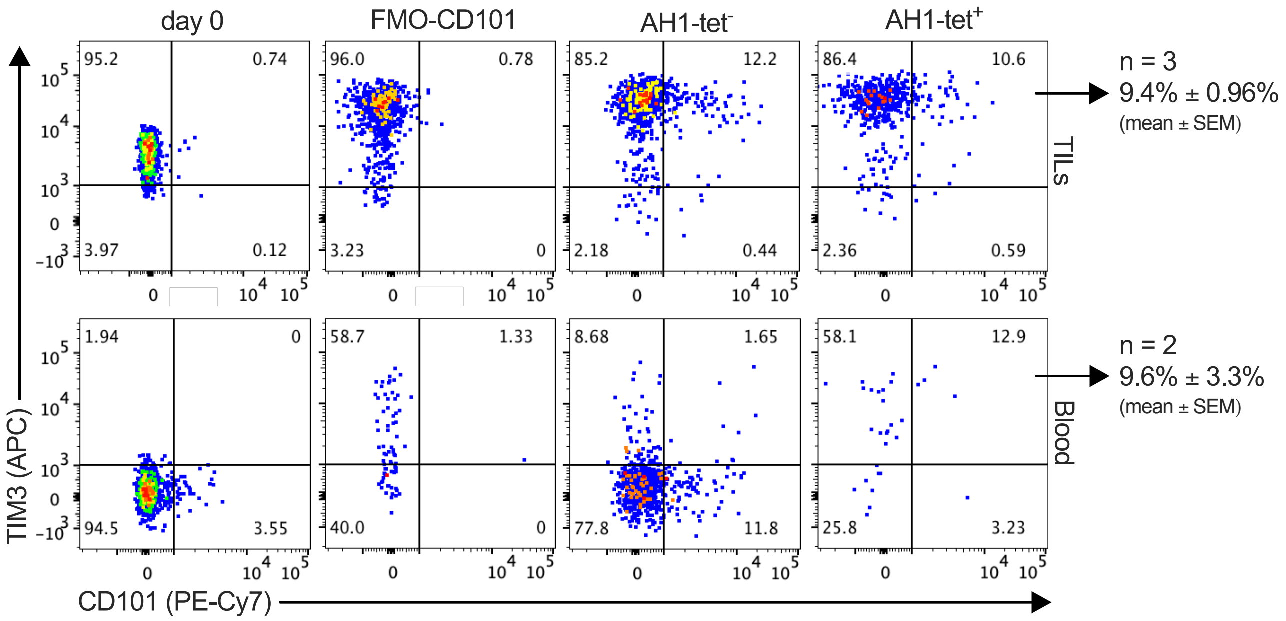

Supplementary Figure 6. Conversion of CD101$^-$ to CD101$^+$ CD8$^+$ T cells. CD8$^+$ T cells were isolated from tumor and blood single-cell suspensions of RT + αPD1 + IL-2c-treated C51 tumor-bearing mice, and CD101$^-$TIM3$^+$PD1$^+$ (from the tumor) or CD101$^-$TIM3$^-$PD1$^+$ (from peripheral blood) CD8$^+$ T cells were flow-cytometrically sorted. The sorted CD8$^+$ T cells were then incubated with IFNγ-pretreated C51 tumor cells at a ratio of 1:5. After 3 days, TIM3 and CD101 was determined on tetramer$^+$ and on tetramer$^-$ CD8+ T cells. One of two experiments using cells from different mice with similar result is shown. Source data are provided as a Source Data file.

Supplementary Figure 7

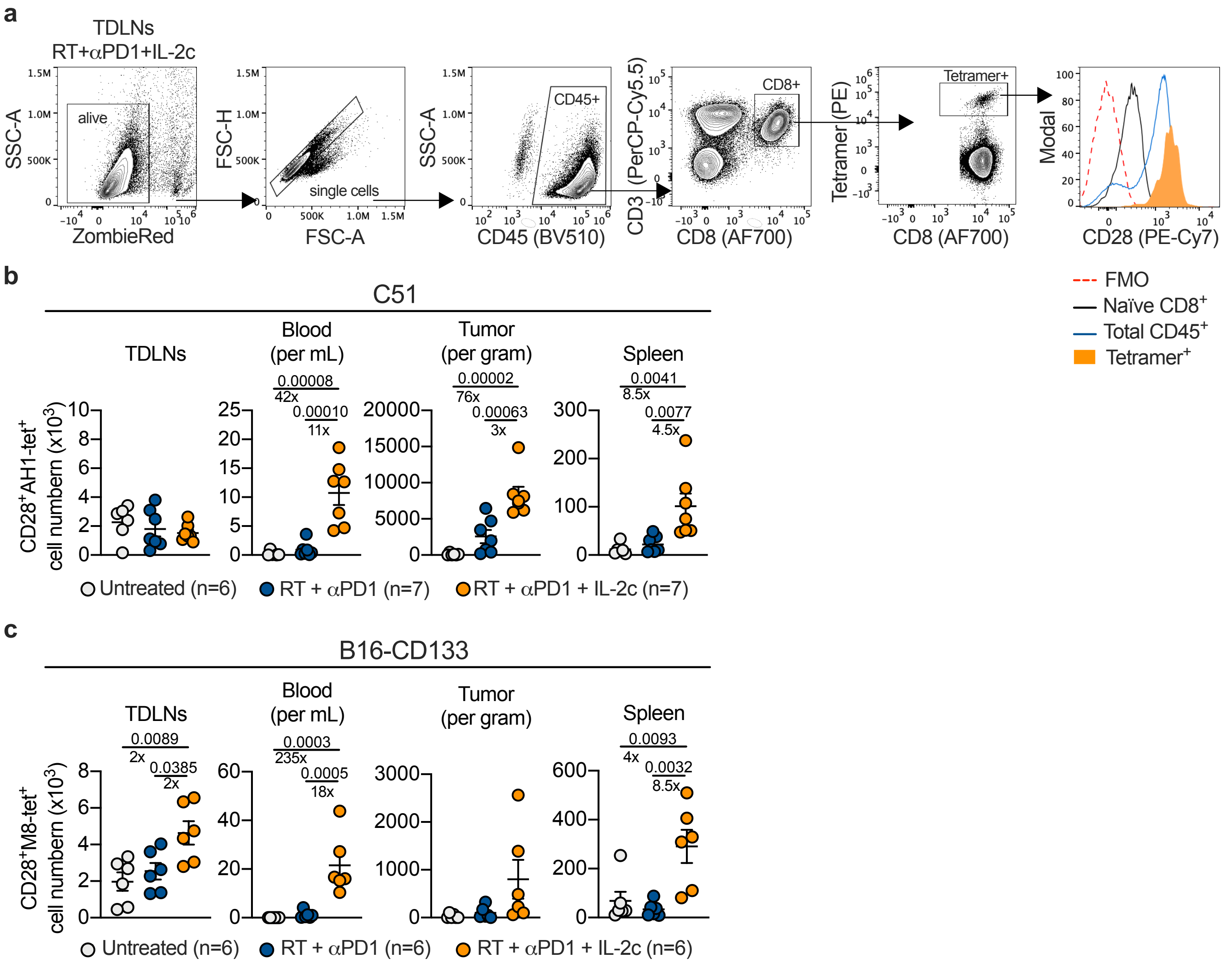

Supplementary Figure 7. Increased CD28⁺ tumor-specific CD8⁺ T cells after RT + αPD1 + IL-2c therapy.

a, Gating strategy to determine CD28⁺ tumor-specific T cells. **b, c,** Numbers of CD28⁺tetramer⁺ cells in TDLNs, blood, tumor and spleen of mice treated with RT + αPD1 (n = 6–7), RT + αPD1 + IL-2c (n = 6–7) or untreated mice (n = 6) bearing C51 (**b**) or B16-CD133 (**c**) tumors. Data are presented as mean ± SEM and were collected from three independent experiments. Symbols represent individual mice. *P* values are shown; statistical comparisons were performed using one-way ANOVA with Tukey's multiple comparisons test. Source data are provided as a Source Data file.

Supplementary Figure 8

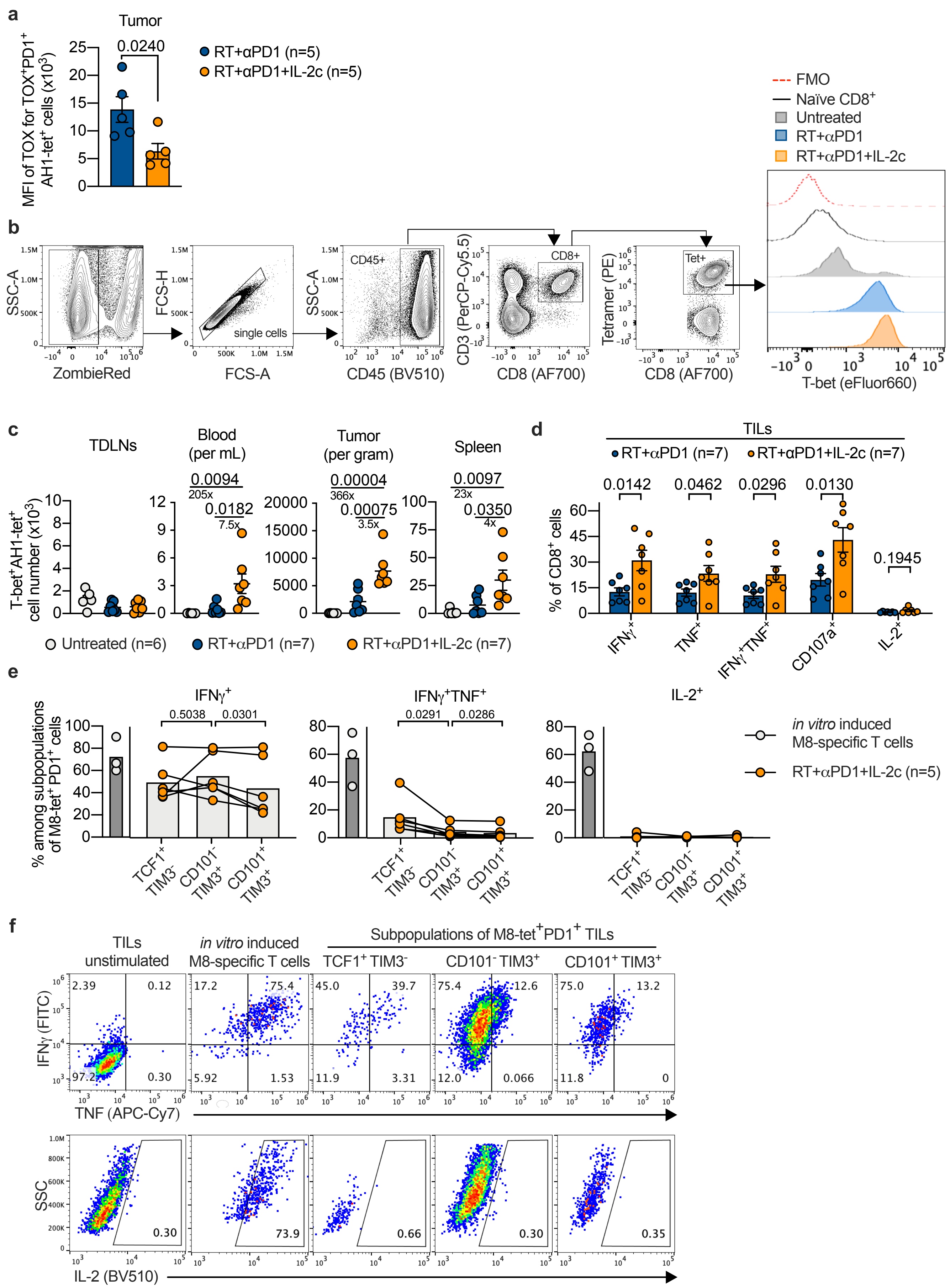

Supplementary Figure 8. Increase in effector-like tumor-specific CD8$^+$ T cells after RT + αPD1 + IL-2c therapy. a, MFI of TOX in tumor-specific PD1$^+$CD8$^+$ T cells in tumors (n = 5). **b,** Gating strategy to identify T-bet$^+$ among tumor-specific CD8$^+$ T cells. **c,** Numbers of T-bet$^+$AH1-tet$^+$ cells in TDLNs, blood, tumor and spleen of mice treated with RT + αPD1 (n = 7), RT + αPD1 + IL-2c (n = 7) or untreated (n = 6) mice bearing C51 tumors. **d,** Percentage of IFNγ$^+$, TNF$^+$, IFNγ$^+$TNF$^+$, CD107a$^+$, and IL-2$^+$ cells among CD8$^+$ T cells isolated at d8 after treatment start and *ex vivo* stimulated with AH1 peptide. Data are presented as mean ± SEM and were collected from three independent experiments. Symbols represent individual mice. **e, f,** Percentage (**e**) and representative flow cytometric plots (**f**) of cytokine-producing cells among different subpopulations of M8-tet$^+$PD1$^+$ TILs of RT + αPD1 + IL-2c-treated mice bearing B16-CD133 tumors (n = 6 individual mice pooled from 3 individual experiments) or M8-specific T cells induced *in vitro* (n = 3 wells). *P* values are shown; statistical comparisons were performed using two-tailed unpaired Student's t test (a, d), one-way ANOVA with Tukey's multiple comparisons test (c), or two-tailed paired Student's t test (e). Source data are provided as a Source Data file.

Supplementary Figure 9

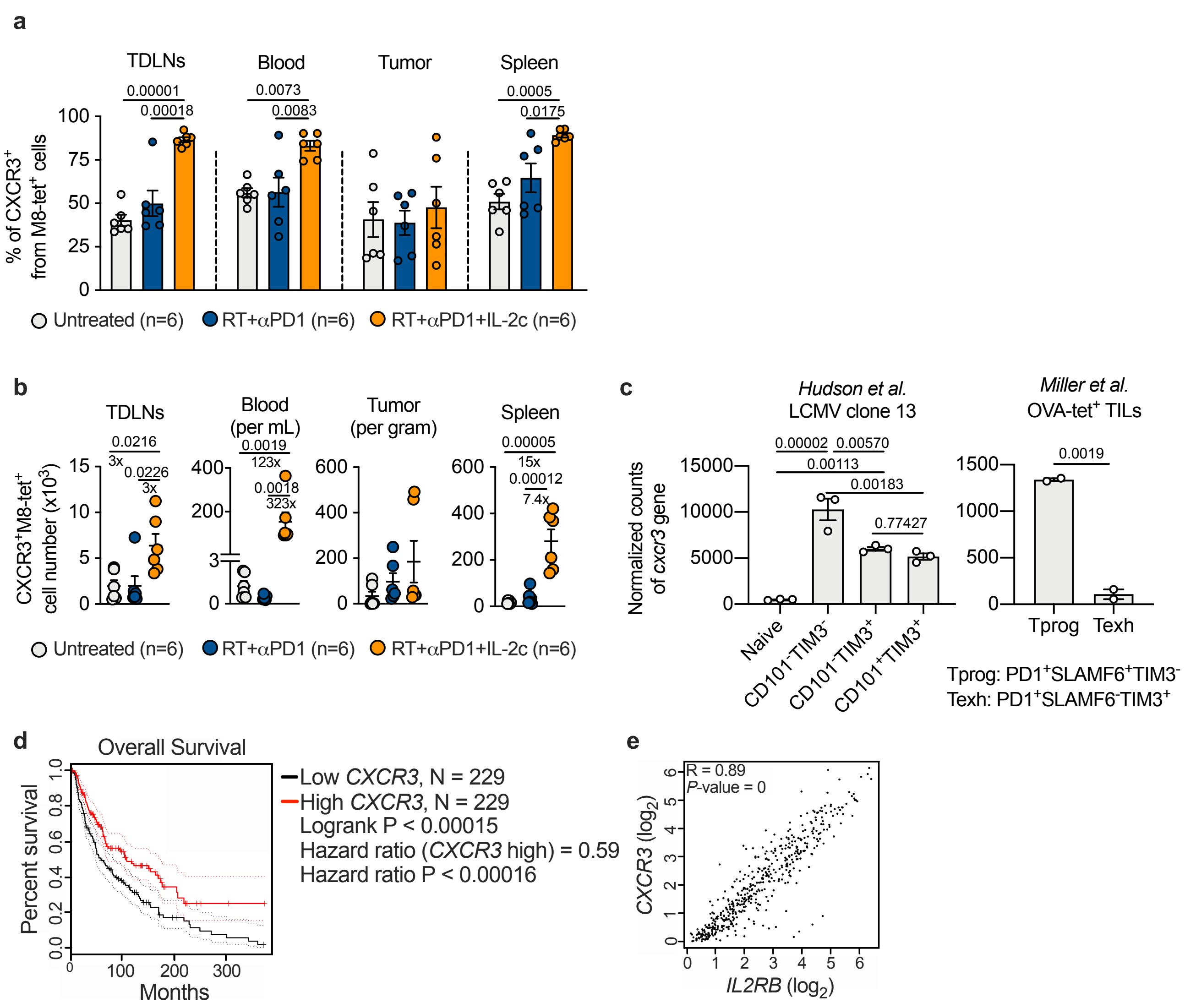

Supplementary Figure 9. Increase in CXCR3$^+$tet$^+$ T cells and their stem-like subset after adding IL-2c to RT + αPD1, and correlation between CXCR3 expression and survival in melanoma patients. a-b, Percentage (**a**), and numbers (**b**) of CXCR3$^+$M8-tet$^+$ cells in the B16-CD133 model (n = 6 mice). Data are presented as mean ± SEM and were collected from three independent experiments. **c,** Normalized counts (RNA-seq from publicly available datasets) of CXCR3 gene expression at different CD8$^+$ T cell differentiation stages in LCMV clone 13 (Hudson *et al.*) and B16-OVA melanoma (Miller *et al.*) models; error bars represent SEM. **d,** Survival analysis of skin cutaneous melanoma (SKCM) patients from TCGA according to *cxcr3* gene expression (cut-off at 50%). **e,** Correlation of *CXCR3* with *IL2RB* gene expression in tumors of SKCM patients. *P* values are shown; statistical comparisons were performed using one-way ANOVA with Tukey's multiple comparisons test (a, b, c left panel), or two-tailed unpaired Student's t test (c right panel), log-rank test (d), or two-tailed Pearson correlation coefficients (e). Source data are provided as a Source Data file.

Supplementary Figure 10

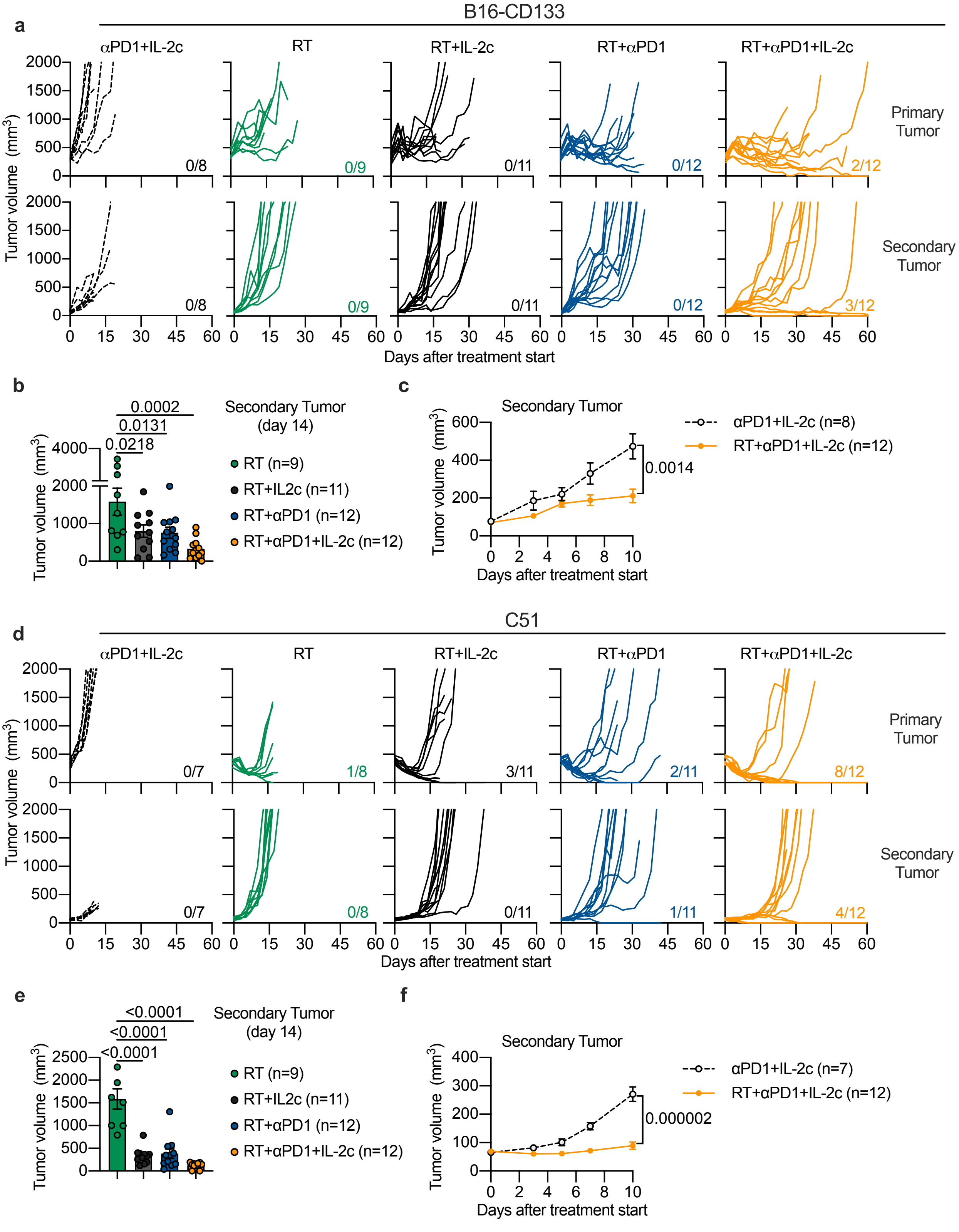

Supplementary Figure 10. Local and abscopal effects of RT combined with IL-2c and αPD-1. a, d, Individual tumor growth curves of primary (upper) and non-irradiated secondary (lower) B16-CD133 (**a**) and C51 (**d**) tumors; numbers in the right lower corner indicate how many tumors were cured. **b, e,** Comparison of the volume of secondary tumors between RT and combination treatment groups at d14 after treatment start in the B16CD133 (**b**) and the C51 (**e**) tumor model; symbols represent individual mice. **c, f,** B16-CD133 (**c**), and C51 (**f**) growth of non-irradiated secondary tumors of mice treated with αPD1 + IL-2c or RT + αPD1 + IL-2c. Data are presented as mean with SEM and were collected from 2-3 independent experiments. Number of mice in each group is indicated in the figure. *P* values are shown; statistical comparisons were performed using one-way ANOVA with Dunnet's multiple comparisons test (b, e), or two-tailed unpaired Student's t-test (c, f). Source data are provided as a Source Data file.

Supplementary Figure 11

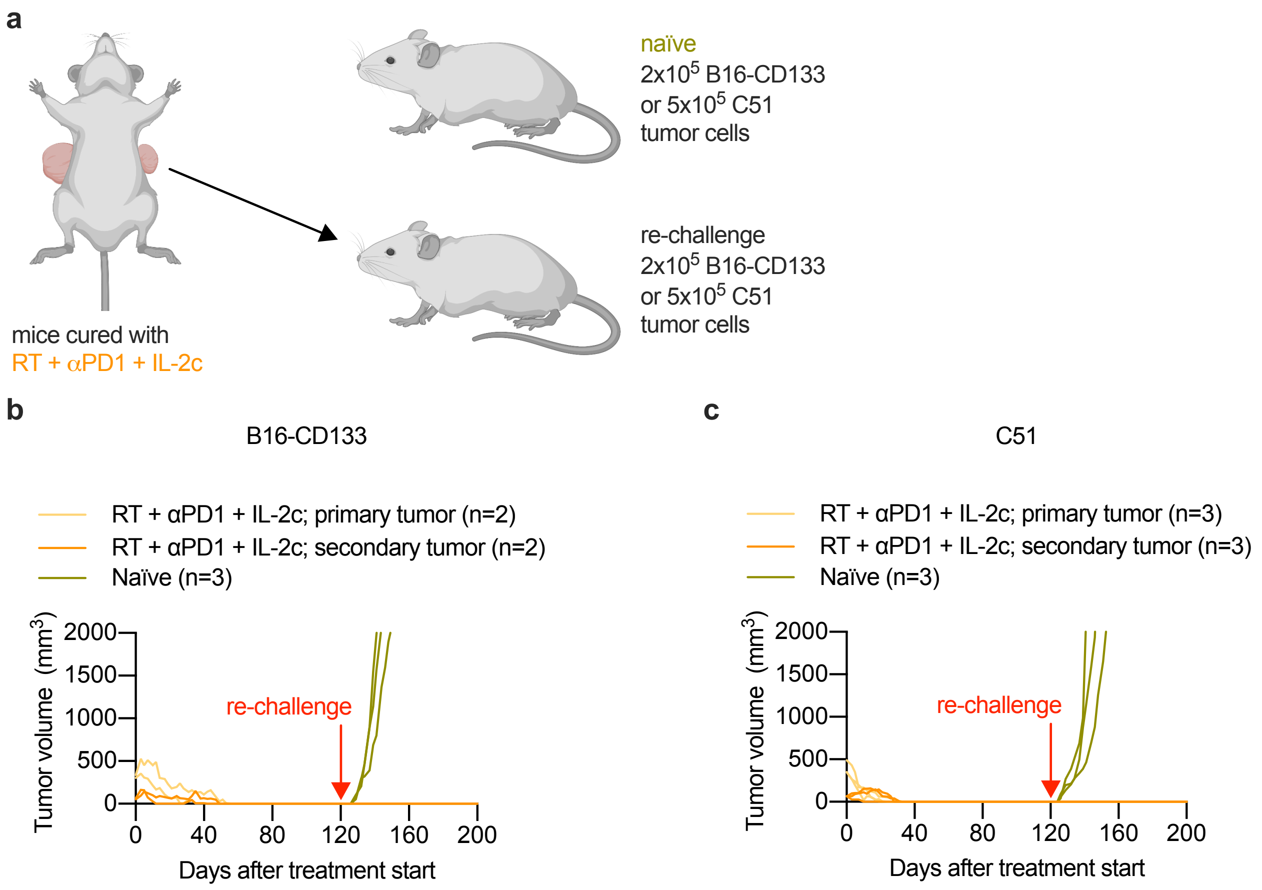

Supplementary Figure 11. Mice cured after RT + αPD1 + IL-2c treatment developed memory against B16-CD133 and C51 tumors. a, Scheme of re-challenge experiment. **b-c**, Individual tumor growth curves of naïve (n = 5) and cured mice (B16-CD133, n = 2; C51, n = 3) challenged with $2x10^5$ B16-CD133 (**b**) or $5x10^5$ C51 (**c**) tumor cells. Source data are provided as a Source Data file.

Supplementary Figure 12

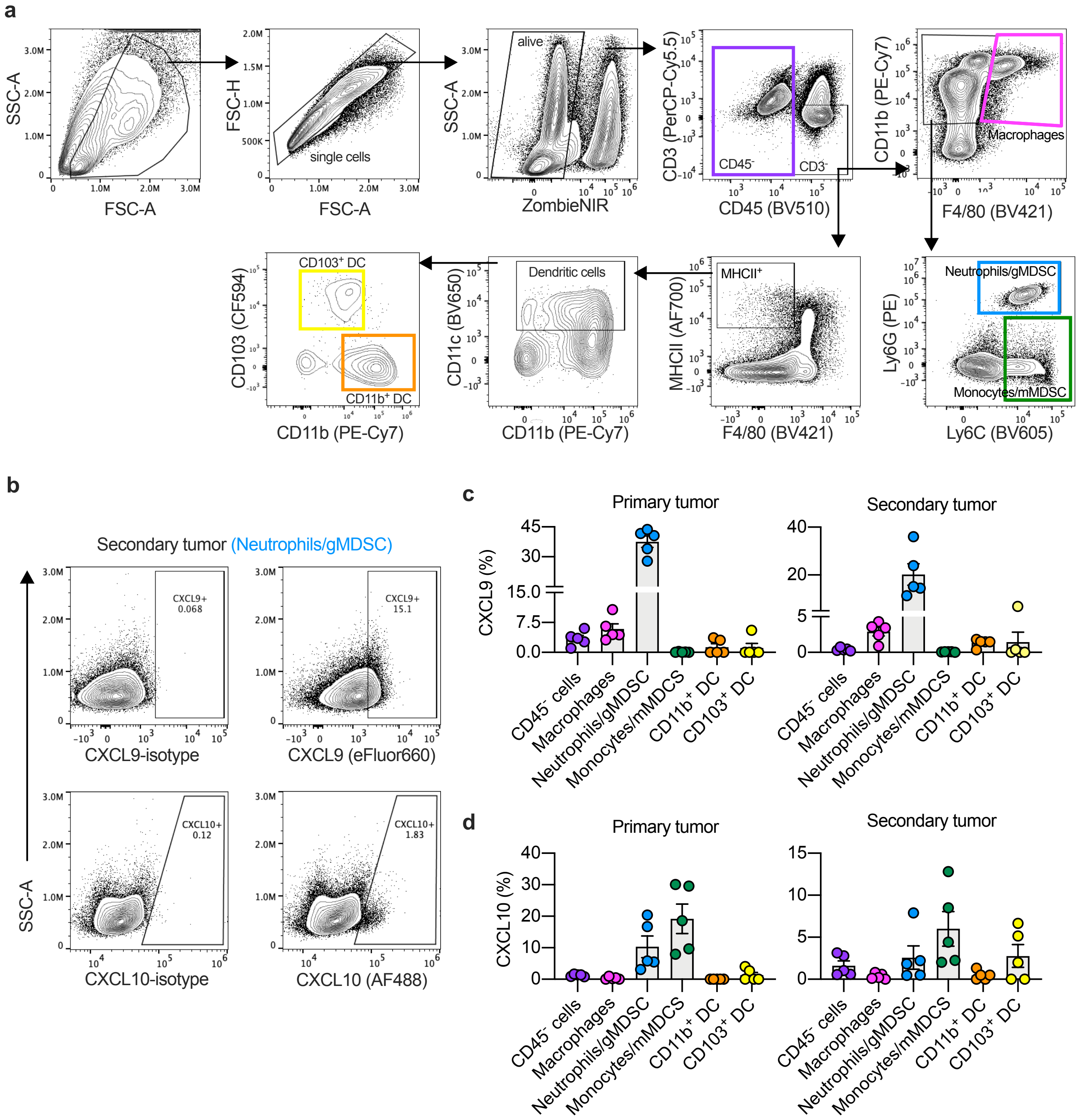

Supplementary Figure 12. Myeloid cells are main producers of CXCL9 and CXCL10 in both irradiated and non-irradiated tumor after RT + αPD1 + IL-2c treatment. a, Gating strategy for identification of $CD45^-$ cells and $CD45^+$ subpopulations of myeloid-lineage cells. The subpopulations are marked in colors; the following subpopulations were identified: macrophages ($CD45^+CD3^-F4/80^+CD11b^+$), neutrophils/gMDSC ($CD45^+CD3^-F4/80^-CD11b^+Ly6C^+Ly6G^+$), monocytes/mMDSC ($CD45^+CD3^-F4/80^-CD11b^+Ly6C^+Ly6G^-$), and dendritic cells ($CD45^+CD3^-$F4/80-$MHCII^+CD11c^+$ $CD11b^+$ or $CD103^+$). **b,** Representative stainings and isotype controls of intracellular flow-cytometric analysis of CXCL9 and CXCL10 following i.v. injection of brefeldin A into RT + αPD1 + IL-2c-treated mice for 12h. **c, d,** Percentage of CXCL9- (**c**) and CXCL10- (**d**) secreting cells in $CD45^-$ and subpopulations of myeloid-lineage cells (n = 5 mice). Data are presented as mean ± SEM. Source data are provided as a Source Data file.

Supplementary Figure 13

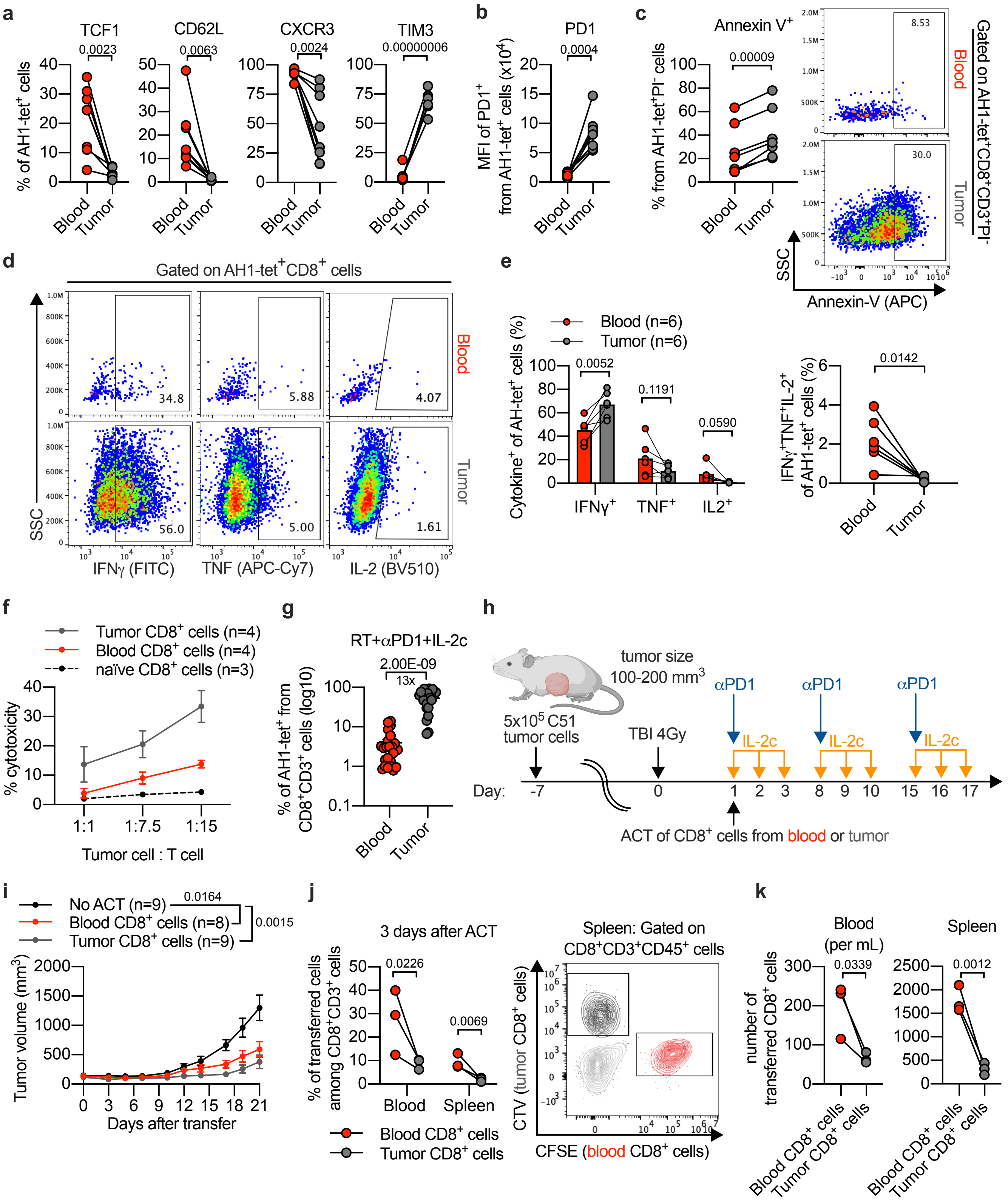

Supplementary Figure 13. Adoptive transfer of CD8$^+$ T cells isolated from blood or tumors of RT + αPD1 + IL-2c-treated mice in the C51 model. a, b, Flow cytometry analysis of AH1-tet$^+$ CD8$^+$ T cells from blood vs. tumor at d8 after start of RT + αPD1 + IL-2c treatment (n = 8). **c**, Flow cytometry analysis and representative example of the early apoptotic (Annexin-V$^+$) population among AH1-tet$^+$PI$^-$ CD8$^+$ T cells in blood and tumor (n = 7). **d-e**, Representative flow cytometry plots (**d**) and percentage (**e**) of IFNγ$^+$, TNF$^+$, IL-2$^+$ (left), and IFNγ$^+$TNF$^+$IL-2$^+$ polyfunctional cells (right) among AH1-tet$^+$CD8$^+$ T cells from blood and tumor after 4h *ex vivo* stimulation with PMA plus ionomycin (n = 6). **f**, *ex vivo* cytotoxicity assay: CD8$^+$ T cells isolated from tumor and blood of triple-treated mice (n = 4) or from spleens of naïve mice (n = 3) were co-incubated with C51 tumor cells at different ratios. **g**, Comparison of the percentage of AH1-tet$^+$ among CD8$^+$ T cells in blood vs. tumor of mice treated with RT + αPD1 + IL-2c at d8 after treatment start (n = 23). **h**, Scheme of adoptive T cell transfer (ACT) experiment in the C51 tumor model. **i**, C51 tumor growth in recipient mice after ACT of CD8$^+$ T cells isolated from blood (n = 8) or tumors (n = 9) of donor mice, or without cell transfer (n = 9). **j**, **k**, Detection of transferred donor T cells in blood and spleen of recipient mice at d7 after ACT. Donor T cells from blood were labeled with CFSE dye and donor T cells from tumors were labeled with CTV dye. Percentage (**j**, left**),** representative flow cytometry plot (**j**, right), and absolute numbers (**k**) of detected transferred donor T cells. Data were collected from 2-3 independent experiments (a-e), or one of two independent experiments are shown (f). Symbols represent individual mice (a-e, j-k). *P* values are shown; statistical comparisons were performed using two-tailed paired Student's t-test (a-e, j-k), or one-way ANOVA with Tukey's multiple comparisons test (f**).** Source data are provided as a Source Data file.

Supplementary Figure 14

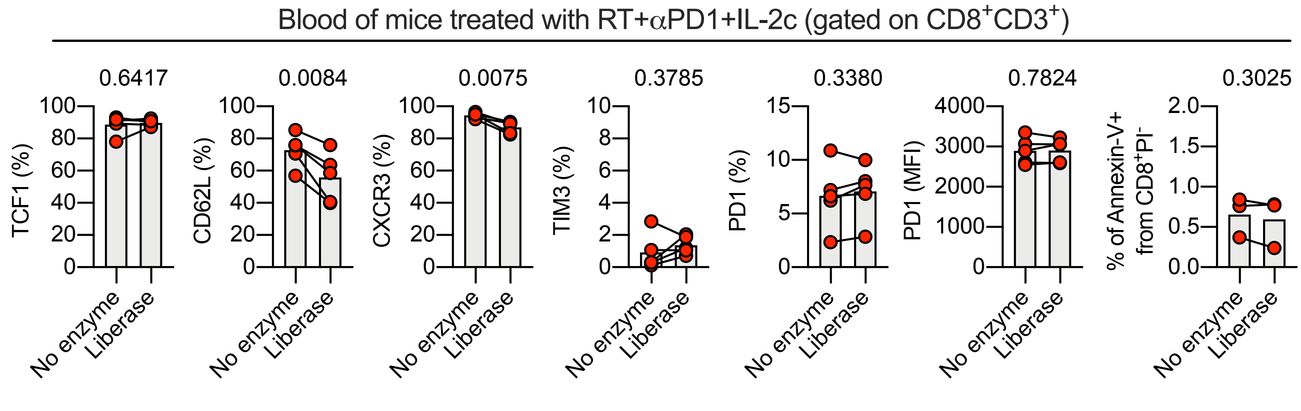

Supplementary Figure 14. Effect of Liberase digestion on surface expression of selected molecules. a, Single-cell suspensions from blood of RT + αPD1 + IL-2c-treated mice (n = 5 mice) were incubated on ice without Liberase or at 37°C in PBS plus $MgCl_2$ plus $CaCl_2$ supplemented with 50 µg/ml DNAse and 120 µg/ml Liberase, the enzyme used to prepare tumor single-cell suspensions. Data were collected from two independent experiments. *P* values are shown and bars indicate mean; statistical comparisons were performed using two-tailed paired Student's t-test. Source data are provided as a Source Data file.

Supplementary Figure 15

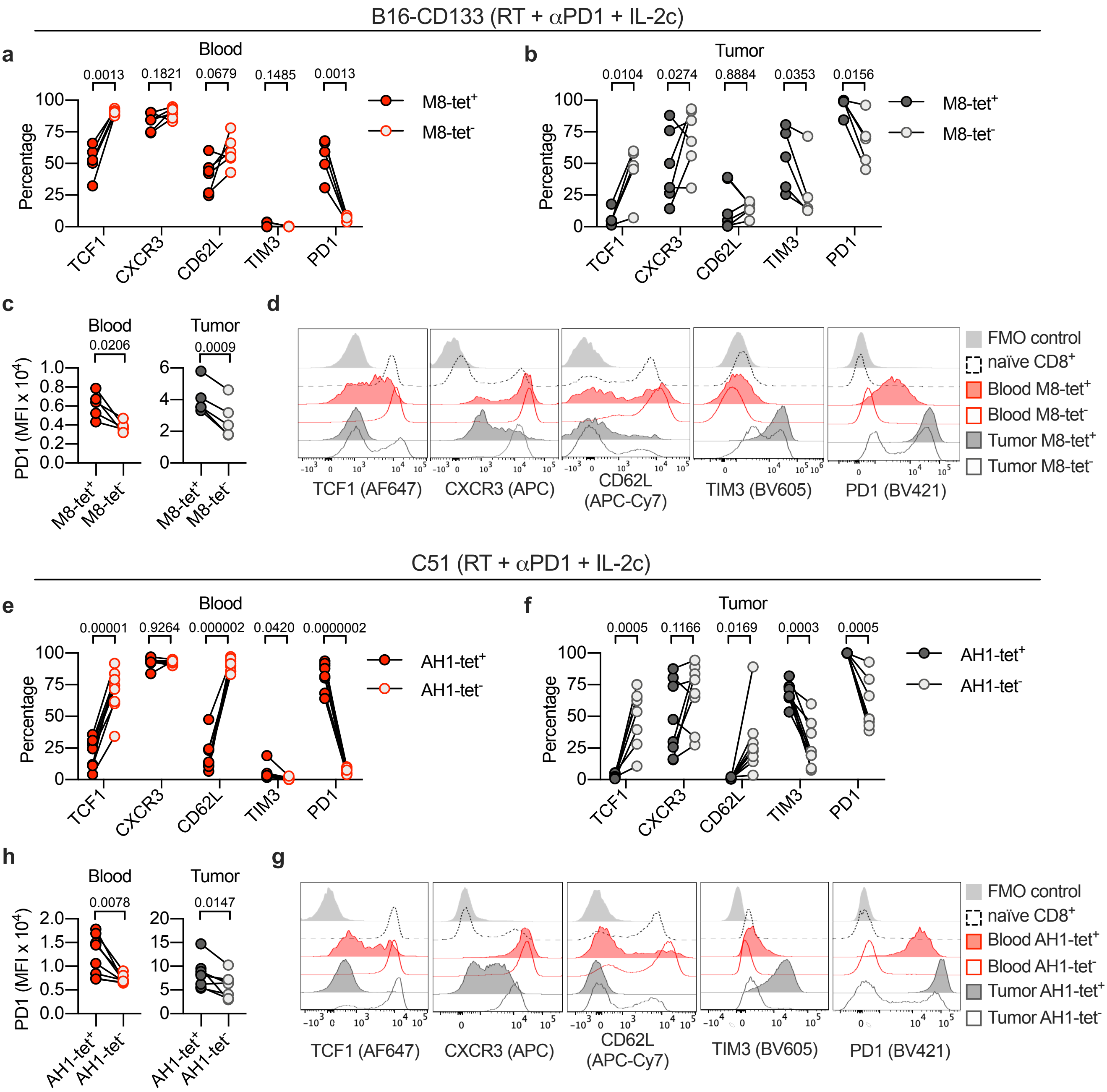

Supplementary Figure 15. Tetramer⁺ T cells in blood and tumor of triple-treated mice had a distinct phenotype compared to tetramer⁻ T cells. a-b, e-f, Percentage comparison of the indicated molecules among tetramer-positive and tetramer-negative $CD8^+$ T cells in blood (**a, e**) and tumors (**b, f**) of B16-CD133 (**a-b**) and C51 (**e-f**) tumor-bearing mice. **c, h,** Comparisons of PD1 expression (MFI) among tetramer-positive and tetramer-negative cells in blood and tumors of B16-CD133 (**c**) and C51 (**h**) tumor-bearing mice. **d, g,** Representative flow cytometry examples. Data were collected from three independent experiments. Symbols represent individual mice. *P* values are shown; statistical comparisons were performed using two-tailed paired Student's t test. Source data are provided as a Source Data file.

Supplementary Figure 16

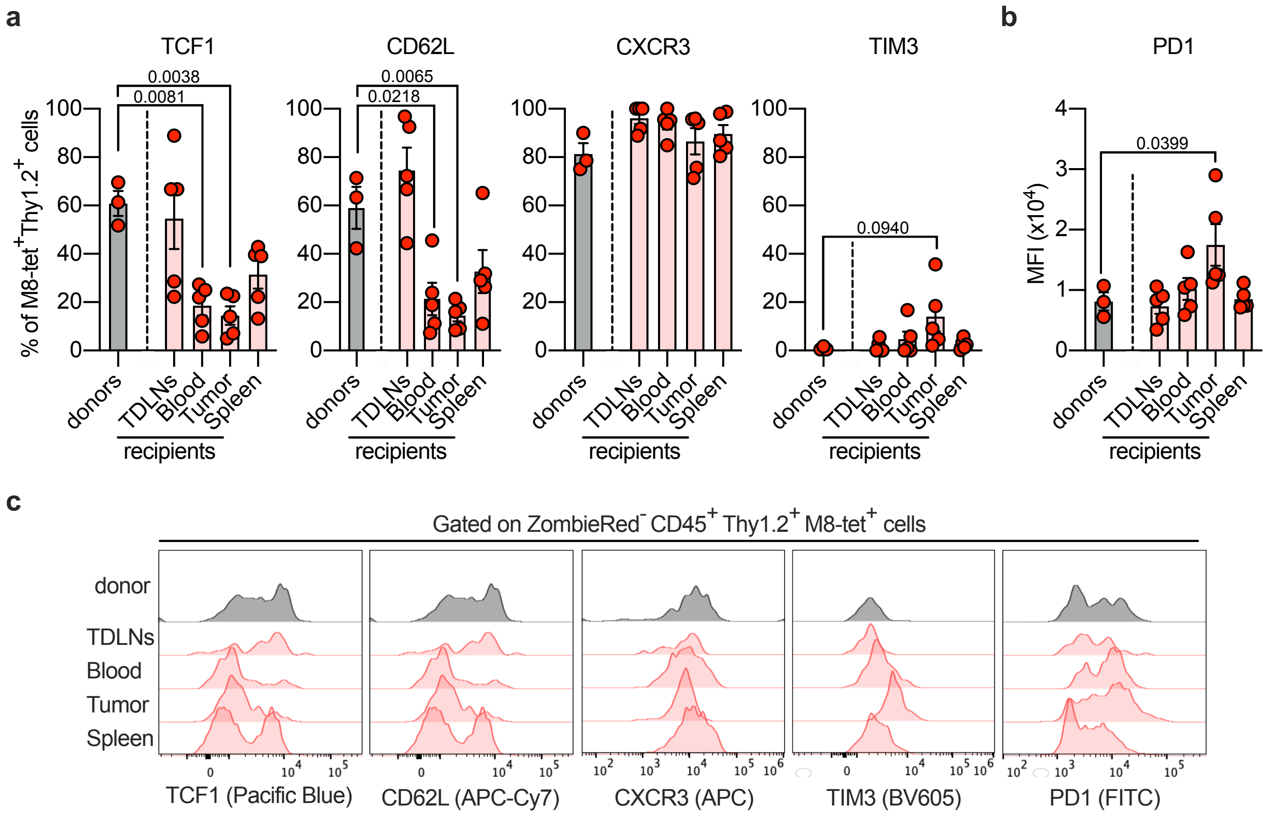

Supplementary Figure 16. Phenotypic changes of donor blood-derived Thy1.2^{+}M8-tet^{+} CD8 T cells 7 days after transfer. Percentage (**a**) or MFI (**b**) of indicated molecules in TDLNs, blood, tumor, and spleen of recipient mice (n = 5) among the transferred M8-tet^{+} CD8^{+} T cells 7 days after the transfer; comparison to the M8-tet^{+}CD8^{+} T cells of donor mice before the transfer (n = 3). **c**, Representative flow cytometry examples. Data are presented as mean ± SEM and were collected from three independent experiments with 1-2 mice each. Symbols represent individual mice. *P* values are shown; statistical comparisons were performed using one-way ANOVA with Dunnet's multiple comparisons test. Source data are provided as a Source Data file.
