# Supplementary material for: Expansion of circulating stem-like CD8+ T cells by adding CD122-directed IL-2 complexes to radiation and anti-PD1 therapies in mice
Source: Nat Commun. 2023 Apr 12;14:2087. doi: 10.1038/s41467-023-37825-x (PMC10097749; doi:10.1038/s41467-023-37825-x)
Supplement: Supplementary file 2 — Reporting Summary [file 41467_2023_37825_MOESM2_ESM.pdf]

nature portfolio

Corresponding author(s): Gabriele Niedermann

Last updated by author(s): Mar 2, 2023

Reporting Summary

Nature Portfolio wishes to improve the reproducibility of the work that we publish. This form provides structure for consistency and transparency in reporting. For further information on Nature Portfolio policies, see our Editorial Policies and the Editorial Policy Checklist.

Statistics

For all statistical analyses, confirm that the following items are present in the figure legend, table legend, main text, or Methods section.

n/a	Confirmed	
☐	☒	The exact sample size (n) for each experimental group/condition, given as a discrete number and unit of measurement
☐	☒	A statement on whether measurements were taken from distinct samples or whether the same sample was measured repeatedly
☐	☒	The statistical test(s) used AND whether they are one- or two-sided<br>*Only common tests should be described solely by name; describe more complex techniques in the Methods section.*
☒	☐	A description of all covariates tested
☐	☒	A description of any assumptions or corrections, such as tests of normality and adjustment for multiple comparisons
☐	☒	A full description of the statistical parameters including central tendency (e.g. means) or other basic estimates (e.g. regression coefficient) AND variation (e.g. standard deviation) or associated estimates of uncertainty (e.g. confidence intervals)
☐	☒	For null hypothesis testing, the test statistic (e.g. F, t, r) with confidence intervals, effect sizes, degrees of freedom and P value noted<br>*Give P values as exact values whenever suitable.*
☒	☐	For Bayesian analysis, information on the choice of priors and Markov chain Monte Carlo settings
☒	☐	For hierarchical and complex designs, identification of the appropriate level for tests and full reporting of outcomes
☐	☒	Estimates of effect sizes (e.g. Cohen's d, Pearson's r), indicating how they were calculated

Our web collection on statistics for biologists contains articles on many of the points above.

Software and code

Policy information about availability of computer code

Data collection	Flow cytometry data was acquired using a CytoFlex S Flow Cytometer (Beckman Coulter) with CytExpert 2.4.0.28 software.<br>Cell sorting was performed using a MoFlo Astrios EQ with Summit v 6.3.1. software.
Data analysis	Data was analysed with Graphpad Prism 8.0 and FlowJo 10.4.<br>For Supplementary Fig. 9d-e, analysis of Cancer Genome Atlas (TCGA) data-base was performed using GEPIA2.0 (http://gepia2.cancer-pku.cn).

For manuscripts utilizing custom algorithms or software that are central to the research but not yet described in published literature, software must be made available to editors and reviewers. We strongly encourage code deposition in a community repository (e.g. GitHub). See the Nature Portfolio guidelines for submitting code & software for further information.

Data

Policy information about availability of data

All manuscripts must include a data availability statement. This statement should provide the following information, where applicable:

- Accession codes, unique identifiers, or web links for publicly available datasets
- A description of any restrictions on data availability
- For clinical datasets or third party data, please ensure that the statement adheres to our policy

Data are available within the Article and Supplementary Information. Source data are provided with this paper, except for Supplementary Fig. 9c-e. For Supplementary Fig. 9c, publicly available datasets were used from Miller et al. (PMID: 30778252) - accessible through the GEO series accession number GSE122713;

Hudson et al. (PMID: 31810882) - available from the NCBI Sequence Read Archive under BioProject PRJNA497086, and normalized gene counts are from Supplementary Information of the paper (https://doi.org/10.1016/j.immuni.2019.11.002). The publicly available online tool GEPIA2.0 utilized to generate the graphs for Supplementary Fig. 9d-e (http://gepia2.cancer-pku.cn, as indicated in methods) does not make the data available.

Human research participants

Policy information about studies involving human research participants and Sex and Gender in Research.

Reporting on sex and gender	n/a
Population characteristics	n/a
Recruitment	n/a
Ethics oversight	n/a

Note that full information on the approval of the study protocol must also be provided in the manuscript.

Field-specific reporting

Please select the one below that is the best fit for your research. If you are not sure, read the appropriate sections before making your selection.

[x] Life sciences [ ] Behavioural & social sciences [ ] Ecological, evolutionary & environmental sciences

For a reference copy of the document with all sections, see nature.com/documents/nr-reporting-summary-flat.pdf

Life sciences study design

All studies must disclose on these points even when the disclosure is negative.

Sample size	Previous experience served as the basis for calculations of expected averages and deviations used to calculate sample size to detect an effect with 80% power. This typically resulted in a minimum sample size of n = 5–7. For some experiments with high variability, sample size was increased.
Data exclusions	In tumor growth experiments, mice with symptoms not attributable to cancer were excluded according to animal welfare criteria. In flow cytometry experiments, all data were included unless there was low cell viability after single cell preparation or unless there were technical issues.
Replication	Data were collected from at least 2-3 independent experiments as indicated in the figure legends. The experiments in Figs. 7f, k, i, Suppl. Fig. 6, Suppl. Fig. 13f, i-k show one of two similar independent experiments. Re-challenge experiments in Suppl. Fig. 1 and Suppl. Fig. 11 were done once.
Randomization	Age and sex-matched mice with similar (predefined) tumor sizes were randomized to the different treatment groups.
Blinding	Since mice were already randomized to maintain a similar average tumor size before treatment in all experimental groups and the local animal care committee requests detailed cage labeling, investigators were not blinded to group allocation.

Reporting for specific materials, systems and methods

We require information from authors about some types of materials, experimental systems and methods used in many studies. Here, indicate whether each material, system or method listed is relevant to your study. If you are not sure if a list item applies to your research, read the appropriate section before selecting a response.

Materials & experimental systems

n/a	Involved in the study	
[ ]	[x]	Antibodies
[ ]	[x]	Eukaryotic cell lines
[x]	[ ]	Palaeontology and archaeology
[ ]	[x]	Animals and other organisms
[x]	[ ]	Clinical data
[x]	[ ]	Dual use research of concern

Methods

n/a	Involved in the study	
[x]	[ ]	ChIP-seq
[ ]	[x]	Flow cytometry
[x]	[ ]	MRI-based neuroimaging

Antibodies

Antibodies used

InVivoMAb:
Anti-PD1 (clone RMP1-14; Cat No. BE0146), anti-CXCR3 (clone CXCR3-173; Cat No. BE0249), and anti-CD40 (clone MR-1; Cat. No. BE0017-1) were purchased from BioXcell.
Anti-CD8-beta antibodies were produced in the lab from the hybridoma clone YTS 156.7.7 purchased from ECACC.
S4B6 anti-mIL-2 antibodies were produced in the lab from the hybridoma HB-10968™ purchased from ATCC.

For flow cytometry analysis, the following antibodies and MHC tetramers were purchased from:

Baylor College of Medicine (Houston, TX):
M8 tetramer – PE (H-2Kb, p15E, KSPWFTTL; 1:200),
AH1 tetramer – PE (H2Ld, gp70, SPSYVYHQF; 1:200),
OVA tetramer – APC (H-2Kb, SIINFEKL; 1:200),
Ag85A tetramer – APC (H-2Ld, Mtb, MPVGGQSSF; 1:200);

BioRad:
CD8 – AF700 (clone KT15, Cat No. MCA609G; 1:50);

BioLegend:
CD45 – BV510 (clone 30-F11, Cat No. 103138; 1:200),
CD3e – PerCP-Cy5.5 (clone 145-2C11, Cat No. 100328; 1:200),
PD1 – BV421 (clone 29F.1A12, Cat No. 135218; 1:200),
PD1 – FITC (clone 29F.1A12, Cat No. 135214; 1:200),
TIM3 – BV605 (clone RMT3-23, Cat No. 119721; 1:200),
CD101 – PE-Cy7 (clone Moushi101, Cat No. 331014; 1:200),
CD69 – FITC (clone H1.2F3, Cat No. 104505; 1:200),
CD28 – PE-Cy7 (clone 37.51, Cat No. 102126; 1:200),
CXCR3 – APC-Fire750 (clone CXCR3-173, 126540; 1:200),
CD62L-APC-Cy7 (clone MEL-14, Cat No. 104428; 1:200),
TNF-alpha – BV421 (clone MP6-XT22, Cat No. 506328; 1:200),
CD107a – BV421 (clone 1D4B, Cat No. 121618; 1:200),
Ki67 – BV605 (clone 16A8, Cat No. 652413; 1:200),
IL-2 – APC (clone JES6-5H4, Cat No. 503810; 1:200),
IL-2 – BV510 (clone JES6-5H4, Cat No. 503833; 1:200),
CD49b - FITC (clone DX5, Cat No. 108905; 1:200)
F4/80 - BV421 (clone BM8, Cat No. 123131; 1:200),
CD11b - PE-Cy7 (clone M1/70, Cat No. 101215; 1:200),
Ly6C - PE (clone HK1.4, Cat No. 128007; 1:200),
Ly6C - BV605 (clone HK1.4, Cat No. 128035; 1:200),
Thy1.1 - PerCP (clone OX-7, Cat No. 202512; 1:200),
Thy1.2 - PerCP-Cy5.5 (clone 53-2.1, Cat No. 140321; 1:200),
Thy1.2 - APC (clone 53-2.1, Cat No. 140311; 1:200);

eBiosciences/Invitrogen:
CD3e – APC-eFlour780 (clone 145-2C11, Cat No. 47-0031-82),
CD122 – FITC (clone TM-b1, Cat No. 11-1222-82; 1:200),
T-bet – eFlour660 (clone 4B10, Cat No. 50-5825-82; 1:200),
IFN-gamma – FITC (clone XMG1.2, Cat No. 11-7311-82; 1:200),
TOX – eFluor660 (clone TXRX10, Cat No. 50-6502-82; 1:200),
GR1 - APC (clone RB6-8C5, Cat No. 17-5931-82; 1:200);
CXCL9 - eFluor660 (clone MIG-2F5.5, Cat No. 50-3009-80; 1:100)
Armenian Hamster IgG Isotype Control - eFluor660 (clone eBio299Arm, Cat No. 50-4888-82; 1:100),
F(ab')2-Rabbit anti-Goat IgG (H+L) Cross-Adsorbed Secondary Antibody - Alexa Fluor 488 (polyclonal, Cat No. A-21222; 1:100)

BD Biosciences:
CD11c - BV650 (clone HL3, Cat No. 564079; 1:200),
CD103 - PE-CF594 (clone M290, Cat No. 565849; 1:200),
Ly6G - PE (clone 1A8, Cat No. 561104; 1:200);

Miltenyi Biotec:
CXCR3 – APC (clone REA724, Cat No. 130-110-971; 1:50),
TIM3 – APC (clone REA602, Cat No. 130-119-796; 1:50)

Cell Signaling:
TCF1 – Alexa Flour 647 (clone C63D9; Cat No. 6709S; 1:200),
TCF1 – Pacific Blue (clone C63D9; Cat No. 9066S; 1:200).

R&D systems:
CXCL10 - unconjugated (polyclonal, Cat No. AF-466-NA; 0.2mg/ml, 1:25)
Goat IgG Control (polyclonal, Cat No. AB-108-C; 0.2mg/ml, 1:25)

Validation

All antibodies were validated by the manufacturers. The specificity of the M8 and the AH1 tetramers was additionally validated using T cells from naïve mice (Suppl. Fig. 3a-b). CXCR3 blockage was confirmed by in vivo administration of the CXCR3-blocking antibody followed by flow cytometric analysis of CXCR3 expression on T cells from tail blood; CD8 depletion was confirmed by in vivo administration of the CD8 cell-depleting antibody followed by flow cytometric analysis of CD8+ T cells from tail blood.

Statement from BioXcell: https://bioxcell.com/in-vivo-antibodies/invivoplus-antibodies
Our InVivoPlus™ antibodies feature all the great qualities of our InVivoMAb™ antibodies. The InVivoPlus™ versions of our products are structurally and functionally identical to the InVivoMAb™ versions with the added benefit of additional QC measures. InVivoPlus™ antibodies are screened for murine pathogens using ultrasensitive qPCR, screened for protein aggregation via dynamic light scattering, feature advanced binding validation via flow cytometry, ELISA, and/or Western blot, and are guaranteed to contain less than 1 endotoxin unit per milligram. Our InVivoPlus™ line of antibodies are designed to exceed the strict demands and rigorous standards required for in vivo work at any research organization.

Statement from Baylor College of Medicine: https://www.bcm.edu/research/atc-core-labs/mhc-tetramer-production-core/order-guidelines
Quality control of tetramer reagents produced includes the following:
All tetramers will be purified by size exclusion and ion exchange chromatography. FPLC chromatograms of the reagent demonstrating proper folding and multimerization will be recorded.
Each tetramer is tested for the level of biotinylation.
ELISA using anti b2-microglobulin antibodies specific for properly folded MHC I.

Statement from BioLegend: https://www.biolegend.com/en-us/quality/quality-control
Specificity testing of 1-3 target cell types with either single- or multi-color analysis (including positive and negative cell types). Once specificity is confirmed, each new lot must perform with similar intensity to the in-date reference lot. Brightness (MFI) is evaluated from both positive and negative populations. Each lot product is validated by QC testing with a series of titration dilutions.

Statement from Invitrogen: https://www.thermofisher.com/de/de/home/life-science/antibodies/invitrogen-antibody-validation.html
Invitrogen antibodies are currently undergoing a rigorous two-part testing approach
Part 1—Target specificity verification.
This helps ensure the antibody will bind to the correct target. Our antibodies are being tested using at least one of the following methods to ensure proper functionality in researcher's experiments. Click on each testing method below for detailed testing strategies, workflow examples, and data figure legends.
Knockout—expression testing using CRISPR-Cas9 cell models
Knockdown—expression testing using RNAi to knockdown gene of interest
Independent antibody verification (IAV)—measurement of target expression is performed using two differentially raised antibodies recognizing the same protein target
Cell treatment—detecting downstream events following cell treatment
Relative expression—using naturally occurring variable expression to confirm specificity
Neutralization—functional blocking of protein activity by antibody binding
Peptide array—using arrays to test reactivity against known protein modifications
SNAP-ChIP™—using SNAP-ChIP to test reactivity against known protein modifications
Immunoprecipitation-Mass Spectrometry (IP-MS)—testing using immunoprecipitation followed by mass spectrometry to identify antibody targets
Part 2—Functional application validation.
These tests help ensure the antibody works in a particular application(s) of interest, which may include (but are not limited to): Western blotting, Flow cytometry, ChIP, Immunofluorescence imaging, Immunohistochemistry

Statement from BD Biosciences: https://www.bdbiosciences.com/en-us/products/reagents/flow-cytometry-reagents/research-reagents/quality-and-reproducibility
Antibody specificity.
BD Biosciences identifies key targets of interest in scientific research and develops its own specific antibodies or collaborates with top research scientists around the world to license their antibodies. We then transform these antibodies into flow cytometry reagents by conjugating them to a broad portfolio of high-performing dyes, including our vastly popular portfolio of BD Horizon Brilliant™ Dyes.
A world-class team of research scientists helps ensure that these reagents work reliably and consistently for flow cytometry applications.
The specificity is confirmed using multiple methodologies that may include a combination of flow cytometry, immunofluorescence, immunohistochemistry or western blot to test staining on a combination of primary cells, cell lines or transfectant models.
All flow cytometry reagents are titrated on the relevant positive or negative cells. To save time and cell samples for researchers, test size reagents are bottled at an optimal concentration with the best signal-to-noise ratio on relevant models during the product development. To ensure consistent performance from lot-to-lot, each reagent is bottled to match the previous lot MFI. You can look up the Certificate of Analysis and the concentration of test-size human reagents from specific lots via the Concentration Lookup page or BD Regulatory Documents.
Quality control.
Our dedication to rigorous testing and high-quality control standards means that you can use our reagents in your research with the utmost confidence. All BD reagent facilities, including our California Design Center at San Diego, our manufacturing facilities in

Tatabanya (Hungary) and San Diego (USA), and our California instrument facility (Manufacturing and Design Centers) at San Jose, are approved and registered according to the internationally defined ISO 9001 standard.
Once our research and development (R&D) team completes evaluation of a new product, the developed process is transferred to our manufacturing teams, including Quality Control..
Our manufacturing process adheres to standard operating procedures (SOPs) and guidelines, which are based on ISO requirements and are strictly followed, helping ensure that reagents provide consistent results to help give you assurance of experimental success and confidence in your research.
Quality control testing of newly manufactured lots is performed side-by-side with a previously accepted lot as a control, helping to assure that performance of the new lot is both reliable and consistent.

Statement from Miltenyi Biotec: https://www.miltenyibiotec.com/DE-en/products/macs-antibodies/antibody-validation.html
REAfinity Recombinant Antibodies are based on three pillars of validation: reproducibility, specificity, and sensitivity.
1. Antibody reproducibility and consistency.
The nature of our REAfinity Recombinant Antibodies ensures reproducibility since they don't have any Immunoglobulin impurities and don't show background signal due to a mutated Fc region.
Recombinant antibodies ensure high lot-to-lot consistency as compared to traditional hybridoma technology. Mass spectrometry analysis of the purified recombinant antibodies confirms the improved purity of antibody products.
After conjugation of antibodies, any unbound fluorochromes and antibodies are removed to purify the final product.
In addition, all antibodies are tested for lot-to-lot consistency at two stages, during the antibody raw material production as well as the fluorochrome-conjugation process. This includes purification steps to remove unconjugated fluorochromes and antibodies from the mixture, as well as side-by-side comparisons with previous batches.
2. Validation of antibody specificity.
During development of an antibody, a suitable test to verify specificity of the clone is performed. Several approaches are possible: knockout of target protein, epitope competition assay, siRNA knockdown, stimulation of cells, overexpression of target protein, binding to purified antigen (latex bead coating), cross-reactivity.
3. Antibody sensitivity.
Sensitivity of an antibody in an intended application is important for reliable identification of target cells. The following approaches are taken at Miltenyi Biotec to ensure that only functional and validated antibodies are part of the portfolio:
All conjugated antibodies, including multiple conjugates of the same clone, are tested on primary samples. Whenever possible, antibodies are tested in multicolor panels. In addition, antibodies are routinely tested on cells derived from tissues using enzymatic treatment. This allows for validation of antibody sensitivity to epitopes that have undergone enzymatic processing.

Statement from Cell Signaling: https://blog.cellsignal.com/introduction-to-the-hallmarks-of-validation
To ensure our antibodies will work in your experiment, we adhere to the Hallmarks of Antibody Validation™, six complementary strategies that can be used to determine the functionality, specificity, and sensitivity of an antibody in any given assay. CST adapted the work by Uhlen, et. al., ("A Proposal for Validation of Antibodies." Nature Methods (2016)) to build the Hallmarks of Antibody Validation, based on our decades of experience as an antibody manufacturer and our dedication to reproducible science.

Statement from R&D systems: https://www.rndsystems.com/products/rd-systems-approach-antibody-quality
R&D Systems carefully tests every antibody we produce to ensure outstanding performance. Our commitment to quality allows you to be confident in your results and help you generate publication-quality data. Details regarding the steps we take to provide you with only the highest-quality antibodies are listed below.
Quality Control. Each antibody is manufactured under controlled conditions, undergoing rigorous quality control testing to ensure lot-to-lot consistency and outstanding performance in all applications listed on our datasheets.
Extensive Specificity Testing. All antibodies are tested for cross-reactivity with closely related molecules using a variety of applications, including direct ELISA, to ensure specificity. These efforts are facilitated by our extensive library of in-house developed antigens.
Formulation. For maximum stability, most antibodies are supplied lyophilized. This also facilitates shipping and storage.

Eukaryotic cell lines

Policy information about cell lines and Sex and Gender in Research

Cell line source(s)	The C51 colon carcinoma cell line was provided by Dr. Mario Paolo Colombo (Fondazione IRCCS Istituto Nazionale dei Tumori, Milan, Italy). The B16F10 cell line was provided by Prof. Hanspeter Pircher, (Medical Center – University of Freiburg, Germany) and transduced with lentiviral particles encoding the human stem cell marker CD133, and sorted for CD133 expression using the CD133 MicroBead Kit (Miltenyi Biotec).
Authentication	Authentication was performed by parent labs. CD133 expression was regularly confirmed by flow cytometry.
Mycoplasma contamination	The initial vials of cells were expanded to establish a stock which was tested negative for mycoplasma. Only cells from the stock cultured for a limited number of passages were used in the study.
Commonly misidentified lines (See ICLAC register)	None of commonly misidentified lines were used

Animals and other research organisms

Policy information about studies involving animals; ARRIVE guidelines recommended for reporting animal research, and Sex and Gender in Research

Laboratory animals	C57BL/6Nrj and BALB/c mice were purchased from Janvier Labs. C57BL6-Thy1.1 mice were from the local stock of the SPF animal facility of the University of Freiburg. Mice were used at the age of 8-12 weeks at the start of the experiment. Animals were housed under specific pathogen-free conditions with 12 hours light/dark cycle, 21–25°C, 45–65% humidity. A maximum of five mice per cage were housed with unlimited access to food and water.
Wild animals	No wild animals were used in the study.
Reporting on sex	Female mice were used except the experiments with T cell transfer where both male and female mice were used as recipients.
Field-collected samples	No field collected samples were used in the study.
Ethics oversight	All animal experiments were performed in accordance with the German Animal License Regulations and were approved by the animal care committee of the Regierungspräsidium (Federal Ministry for Nature, Environment and Consumers' Protection) Freiburg, Freiburg, Germany (registration numbers: G18/066, G20-016).

Note that full information on the approval of the study protocol must also be provided in the manuscript.

Flow Cytometry

Plots

Confirm that:

- [x] The axis labels state the marker and fluorochrome used (e.g. CD4-FITC).
- [x] The axis scales are clearly visible. Include numbers along axes only for bottom left plot of group (a 'group' is an analysis of identical markers).
- [x] All plots are contour plots with outliers or pseudocolor plots.
- [x] A numerical value for number of cells or percentage (with statistics) is provided.

Methodology

Sample preparation	For single-cell suspension preparation, tumors were weighed and digested in 5 ml of PBS plus MgCl2 plus CaCl2 (Gibco; Thermo Fisher Scientific) supplemented with 120 µg/ml of Blendzyme and 50 µg/ml of DNAse for 20 minutes at 37°C while rotating. Single-cell suspensions were mechanically smashed through 70 µm and filtrated through 30 µm cell strainers (Miltenyi Biotec). The spleens and lymph nodes were squeezed through a 70 µm strainer. Red blood cell lysis was performed using 1X red blood cell lysis buffer (eBioscience).<br>Other experimental details are provided in Materials and Methods.
Instrument	The flow cytometry data was collected by CytoFlex S Flow Cytometer (Beckman Coulter).<br>Cell sorting was performed using MoFlo Astrios EQ (Beckman Coulter).
Software	The flow cytometry data was collected by CytExpert 2.4.0.28 (Beckman Coulter) and analysed by FlowJo 10.4 softwares.<br>Cell sorting was done using Summit v 6.3.1. software.
Cell population abundance	Sorted cells had a purity > 95%, checked by post-sort re-sampling.
Gating strategy	In all samples dead cells were excluded by Propidium Iodide (Miltenyi Biotec, Cat. No. 130-093-233) or ZombieRed/ZombieNIR Fixable Viability dyes (BioLegend, Cat. No. 423109 or 423105), FSC-H/FSC-A gate was used to gate on singlets. CD45+CD3+CD8+ cells were used to determine CD8 T cells, CD45+CD3+CD8+Tetramer+ cells were gated to determine tumor-specific T cells. Further populations (CD122+, Ki67+, CXCR3+, T-bet+, CD28+, TOX+, TCF1-CD69-, TCF1-CD69+, IFN-gamma+, TNF-alpha+, IL-2+, Annexin-V+, CFSE dilution, "stem-like", "transitory", "terminally exhausted") were gated from tumor-specific T cells.<br>TCF1+TIM3-PD1+ cells were used for the identification of the stem-like, CD101-TCF1-TIM3+PD1+ for transitory, and CD101+TCF1-TIM3+PD1+ for terminally exhausted subsets. In Fig. 4e-f, and Suppl. Fig. 8e-f cytokine+, and in Fig. 5d-f CXCR3+ cells were gated among TCF1+TIM3-PD1+, CD101-TCF1-TIM3+PD1+, CD101+TCF1-TIM3+PD1+ subsets.<br>In Fig.4c-d and Suppl. Fig. 8d, cytokine positive cells were gated among CD45+CD3+CD8+ cells.<br>In Fig. 7k, Thy1.1 (recipient) and Thy1.2 (donor) populations, and in Suppl. Fig. 13j, CTV-labeled (tumor-derived CD8+) and CFSE-labeled (blood-derived CD8+) cells were gated from the CD45+CD3+CD8+ population.<br><br>In Suppl. Fig. 2 cells were gated as:<br>CD8 cells: ZombieRed-CD45+CD3+CD8+<br>CD4 cells: ZombieRed-CD45+CD3+CD4+FoxP3-<br>Treg: ZombieRed-CD45+CD3+CD4+CD25+FoxP3+<br>NK cells: ZombieRed-CD45+CD3-CD49b+<br>Neutrophils/gMDSC: ZombieNIR-CD45+CD3-CD11b+F4/80-GR1+

Monocytes/mMDSC: ZombieNIR-CD45+CD3-CD11b+F4/80-GR1-Ly6C+
Macrophages: ZombieNIR-CD45+CD3-CD11b+F4/80+
Dendritic cells: ZombieNIR-CD45+CD3-F4/80-MHC-II+CD11c+

In Suppl. Fig. 12 cells were gated as:
CD45- cells: ZombieNIR-CD45-
Macrophages: ZombieNIR-CD45+CD3-CD11b+F4/80+
Neutrophils/gMDSC: ZombieNIR-CD45+CD3-CD11b+F4/80-Ly6G+Ly6C+
Monocytes/mMDSC: ZombieNIR-CD45+CD3-CD11b+F4/80-Ly6G-Ly6C+
CD11b+ dendritic cells: ZombieNIR-CD45+CD3-F4/80-MHC-II+CD11c+CD11b+
CD103+ dendritic cells: ZombieNIR-CD45+CD3-F4/80-MHC-II+CD103+

☒ Tick this box to confirm that a figure exemplifying the gating strategy is provided in the Supplementary Information.
